# Drug-Loaded Red Blood Cell–Derived Carriers for Targeted Delivery to Accelerate Tissue Regeneration

**DOI:** 10.3390/pharmaceutics18091131

**Published:** 2026-09-09

**Authors:** Kulzhan Berikkhanova, Yernur Zakirov, Askhat Zhilkaidarov, Nurgul Daniyeva, Erlan Taigulov, Saken Kozhakhmetov, Ardak Omarbekov, Beibarys Amankeldin, Gulyash Tanysheva, Nurzhan Berikkhanov, Yessenkhan Sultan, Zhanas Baimagambet, Isah Inuwa, Saule Aliyeva

**Affiliations:** 1 PI «National Laboratory Astana», Nazarbayev University, Astana 010000, Kazakhstan; 2 University Medical Center, Nazarbayev University, Astana 010000, Kazakhstan; 3 Professor G.V. Tsoi Scientific and Educational Center of Surgery, Astana Medical University, Astana 010000, Kazakhstan; 4 Department of Obstetrics and Gynecology Named After A.A. Kozbagarov, Department of Pharmacology Named After MD M.N.Musin, Semey Medical University, Semey 071400, Kazakhstan; 5 City Polyclinic №2, Taraz 080000, Kazakhstan; 6 Medical Center “Sultan-Medicus”, Almaty 050000, Kazakhstan; 7 School of Medicine, Nazarbayev University, Astana 010000, Kazakhstan

**Keywords:** diabetic foot ulcers, targeted drug delivery, epidermal growth factors (EGF), ceftriaxone, tissue regeneration, wound healing

## Abstract

**Background/Objectives:** Diabetic foot ulcers are characterized by persistent inflammation, impaired angiogenesis, and delayed tissue regeneration. Epidermal growth factor (EGF) is an important regulator of wound repair; however, its therapeutic application may be limited by rapid degradation and poor retention within the wound environment. This study investigated the therapeutic efficacy of local administration of EGF and ceftriaxone using autologous red blood cell-derived carriers (RBCDCs) in an experimental model of diabetic wound healing in rats. **Methods:** Experiments were conducted on 30 albino Wistar rats (250 ± 20 g) with experimentally induced diabetic wounds. In the RBCDC treatment groups, separately prepared drug-loaded RBCDC formulations containing EGF or ceftriaxone were locally administered into the wound. The corresponding comparison groups received the same active agents in free form according to an identical treatment schedule, while an additional control group received topical Levomekol. **Results:** RBCDC-mediated administration of EGF and ceftriaxone significantly accelerated wound closure and was associated with earlier resolution of inflammation, granulation tissue maturation, re-epithelialization, collagen deposition, and angiogenesis compared with the corresponding free-drug formulations. On day 9, tissue EGF concentration was higher in the RBCDC (EGF + Ctx) group than in the corresponding free-drug group. Additional formulation characterization demonstrated ceftriaxone encapsulation and in vitro release from RBCDCs, as well as in vitro EGF release from EGF-loaded RBCDCs. **Conclusions:** Autologous RBCDCs represent a promising local drug-carrier platform for the administration of regenerative and antimicrobial agents in experimental diabetic wounds. Further studies are required to establish local pharmacokinetics, systemic exposure, carrier stability, and the mechanisms responsible for the observed therapeutic effects.

## 1. Introduction

Chronic non-healing wounds remain a major clinical challenge, prolonging hospitalization, increasing healthcare costs, and reducing patients’ quality of life [1,2,3]. Diabetic foot ulcers (DFUs) are among the most severe complications of diabetes mellitus and are characterized by persistent inflammation, impaired angiogenesis, metabolic dysregulation, immunosuppression, and bacterial infection, all of which contribute to delayed wound healing [4,5]. Approximately 20–25% of patients with diabetes develop foot ulcers during their lifetime, and nearly one-quarter of these ulcers become infected, frequently leading to lower limb amputation. Despite advances in wound management, DFUs remain associated with substantial morbidity and mortality [4,5].

Normal wound healing is a tightly regulated process involving sequential phases of inflammation, granulation tissue formation, re-epithelialization, extracellular matrix remodeling, and tissue maturation. These processes are coordinated by numerous cytokines and growth factors, including epidermal growth factor (EGF), platelet-derived growth factor (PDGF), vascular endothelial growth factor (VEGF), fibroblast growth factor (FGF), transforming growth factor-β (TGF-β), and inflammatory mediators such as TNF-α, IL-1β, IL-6, IFN-γ, and IL-10 [6,7,8,9,10]. In diabetic and other chronic wounds, prolonged inflammation and dysregulated expression of these mediators impair tissue regeneration, resulting in defective angiogenesis, excessive fibrosis, and delayed wound closure [11,12,13,14,15,16,17].

Among these mediators, EGF plays a central role by stimulating keratinocyte and fibroblast proliferation, collagen synthesis, angiogenesis, and re-epithelialization [18,19,20,21]. Clinical studies have demonstrated that topical or intralesional recombinant human EGF (rhEGF), including Heberprot-P^®^ and Citoprot-P ^®^ accelerates healing of diabetic foot ulcers and reduces amputation rates [22,23,24,25,26]. However, the clinical efficacy of EGF remains limited by its rapid degradation, short plasma half-life, poor retention within the wound environment, and elimination by wound exudate. Consequently, repeated administration or high doses are often required, increasing treatment costs and the potential risk of adverse effects [27,28,29,30,31,32,33].

To overcome these limitations, numerous drug delivery systems, including hydrogels, scaffolds, nanoparticles, liposomes, polymers, and peptide-based carriers, have been developed to improve the stability and local retention of growth factors. Although these systems have shown promising experimental results, their clinical translation is frequently limited by manufacturing complexity, limited biocompatibility, immunogenicity, toxicity, and cost [34,35,36,37,38,39,40].

Autologous red blood cell-derived carriers (RBCDCs) represent a promising physiological platform for drug delivery. Their potential advantages include biocompatibility, biodegradability, immunological compatibility, and the possibility of preparation from autologous blood [41,42,43,44,45,46,47,48].

Drugs can be incorporated into erythrocyte-derived carriers using hypo-osmotic hemolysis and resealing procedures, generating drug-loaded erythrocyte preparations commonly referred to as pharmacocytes. Previous studies have explored erythrocyte-based carriers for the delivery of antibiotics and other therapeutic agents [49,50,51].

Following administration into inflamed tissues, erythrocyte-derived carriers may interact with phagocytic cells, including macrophages, and subsequent degradation of the carrier may contribute to local release of the incorporated therapeutic agent [49,50,51]. However, the extent and kinetics of macrophage-mediated uptake, local drug release, and systemic exposure depend on the specific formulation and route of administration and require direct experimental evaluation.

Based on these considerations, the present study investigated whether local administration of EGF and ceftriaxone using autologous RBC-derived carriers improves healing of experimental diabetic wounds compared with administration of the corresponding free-drug formulations. We evaluated wound closure, cytological and histological changes, collagen deposition, angiogenesis, and tissue EGF concentration, together with additional physicochemical characterization of the drug-loaded RBCDC formulations.

## 2. Materials and Methods

### 2.1. Reagents

Recombinant epidermal growth factor (EGF), (200 μg) and corresponding ELISA kit were procured from Cloud-Clone Corp (Wuhan, China). Ceftriaxone (1 g) were purchased from Sintez (Krasnoyarsk, Russia). Streptozotocin (STZ), (99% 1 g) was purchased from Glentham LIFE SCIENCES, (Corsham SN13 9SW, Wiltshire, UK). Isofluran (250 mL) was purchased from Piramal Enterprises Limited (Mumbai, India). HPLC-grade water, acetonitrile, and methanol and other reagents were all bought from SigmaAldrich Co. (St. Louis, MO, USA). Deionized water was generated by a water purification system. The whole reagents were used following instructions and according to the manufacturer’s protocol.

### 2.2. Experimental Animals

A total of 30 male albino Wistar rats weighing 250 ± 20 g were housed under standard controlled conditions at the National Biotechnology Center’s vivarium. The environmental conditions included a temperature of 22 ± 2 °C, humidity of 55 ± 5%, and a 12-h light/dark cycle. The rats were fed ad libitum with unrestricted access to food and water. The use of experimental animals were approved by the Local Ethical Committee of PI “National Laboratory Astana” (№ 05-2022/21.10.2022) and by the Institutional Animal Care and Use Committee (IACUC) at Nazarbayev University (approval ID: 4/13112024). All procedures were carried out with appropriate anesthesia, under isoflurane inhalation anesthesia.

### 2.3. Preparation of Drug Loaded RBCs-Derived Carriers

RBC-derived carriers (RBCDCs) loaded with selected drugs have been developed using the hypoosmotic hemolysis method, incorporating our original modifications [47,48,49]. For wound treatment, blood samples (1 mL) were collected from the rats’ tail veins, and RBCDCs containing single doses of either EGF (4 μg/kg), or ceftriaxone (100 mg/kg) were prepared using the hypoosmotic hemolysis method, following the pharmacocyte preparation protocol [49,50]. Briefly, to encapsulate drugs into autologous erythrocytes under sterile conditions, 1.0 mL of blood was drawn from the vein into a sterile heparinized tube. After centrifugation at 3000 rpm for 5 min, the supernatant was removed. The precipitate was resuspended in a five-fold volume of physiological saline and washed three times. Distilled water at 0 °C was used to obtain erythrocyte ghosts. After centrifugation, the supernatant was removed. The incubation solution consisted of the investigated drug dissolved in distilled water. Osmotic balance was restored using a 10% sodium chloride solution. EGF and ceftriaxone were loaded into separate RBCDC preparations and were not co-encapsulated within the same carrier formulation. For each treatment session, fresh autologous blood was collected from the corresponding animal and a new drug-loaded RBCDC preparation was produced. The freshly prepared RBCDC formulations were administered immediately after preparation and were not stored. For repeated treatments at 24 and 48 h, blood collection and preparation of the corresponding RBCDC formulations were repeated using newly obtained autologous blood.

#### 2.3.1. Characterization of Ceftriaxone Loading and In Vitro Release

Ceftriaxone encapsulation into erythrocyte-derived carriers was quantified by high-performance liquid chromatography (HPLC) using the previously described procedure [51]. Briefly, ceftriaxone-loaded erythrocytes were centrifuged, and the carrier-containing pellet was collected and lysed in distilled water. The resulting lysate was filtered through a 0.22 μm membrane filter and analyzed by HPLC.

The in vitro release of ceftriaxone from ceftriaxone-loaded RBCDCs was evaluated using a membrane dialysis method as previously described [51]. Ceftriaxone-loaded erythrocytes were transferred into a dialysis membrane and incubated in phosphate-buffered saline (PBS, pH 7.4) at 37 °C with continuous mixing. Samples of the external release medium were collected at predetermined time points and replaced with an equal volume of fresh medium. Ceftriaxone concentrations were quantified by HPLC, and cumulative drug release was expressed as a percentage of the initial ceftriaxone amount.

#### 2.3.2. In Vitro Release of EGF from EGF-Loaded RBCDCs

The in vitro release of EGF from EGF-loaded RBCDCs was evaluated using a dialysis membrane method. One milliliter of EGF-loaded RBCDC suspension was placed into a dialysis membrane and immersed in 15 mL of 0.9% NaCl at 37 °C. Samples of the external dialysis medium were collected at 15 and 30 min and at 1, 1.5, 2, and 3 h. At each sampling point, the external dialysis medium was replaced with an equal volume of fresh 0.9% NaCl. At the end of the experiment, the remaining intramembrane fraction was also collected. EGF concentrations were determined by ELISA after appropriate sample dilution. Six independently prepared EGF-loaded RBCDC samples were analyzed together with control samples.

#### 2.3.3. Particle Size Distribution and Zeta Potential

Particle size and size distribution of EGF-loaded erythrocyte-derived carrier preparations were analyzed by nanoparticle tracking analysis using a ZetaView system (Particle Metrix, Inning am Ammersee, Germany) equipped with a 488-nm laser. Four preparations were analyzed at approximately 25 °C and pH 7.0 after appropriate dilution. Particle-size distributions were characterized by the median diameter (X50), mean diameter, X10, X90, and distribution span. Zeta potential was additionally determined by electrophoretic analysis using the Smoluchowski model.

### 2.4. Preparation of Blood and Tissue Samples for Microscopy

This study utilized standardized protocols for the preparation of blood and tissue samples for scanning electron microscopy (SEM), light microscopy (LM) and transmission electron microscopy (TEM). Morphostructural changes in erythrocytes during drug encapsulation using the hypo-osmotic hemolysis method. The following stages of drug-loaded RBC preparation are shown: 1,2,3—RBCs after plasma removal; 4,5,6—RBCs after three times washing; 7,8,9—hemolysis of RBCs after addition of distilled water; 10,11,12—RBC ghosts after centrifugation; 13,14,15—RBCDCs after incubation with the drug; 16,17,18—drug loaded RBCDCs after “resealing”. The scanning electron microscopy (SEM) images were acquired using an Auriga Crossbeam 540 (Carl Zeiss) equipped with an InLens detector. The transmission electron microscopy (TEM) images were acquired using a JEM-1400Plus (JEOL) in bright-field mode. The light microscopy (LM) images were obtained using a Leica DM4000 light microscope. The magnifications for each panel are specified individually in the figure.

Scanning Electron Microscopy (SEM) Sample Preparation

To study the morphology of blood cells using SEM, blood samples were fixed in 2.5% glutaraldehyde at 4 °C for 24 h and then washed three times in saline to remove residual fixative. Post-fixation was performed using 1% osmium tetroxide for 2 h. Samples were dehydrated in a graded ethanol series (50% to 100%). Dehydrated samples were coated with a 15 nm gold layer and examined using an Auriga Crossbeam 540 scanning electron microscope (Carl Zeiss, AG, Oberkochen, Germany) with an InLens detector at 5 kV.

Light and Transmission Electron Microscopy (LM and TEM) Sample Preparation

Light microscopy (LM) was used to examine the morphological structure of semithin sections of blood and tissue samples, while transmission electron microscopy (TEM) was employed to investigate their ultrastructure. Blood and tissue samples were fixed in 2.5% glutaraldehyde at 4 °C for 24 h and then washed three times in saline to remove residual fixative. Post-fixation was performed using 1% osmium tetroxide for 2 h. Samples were dehydrated in a graded ethanol series (50% to 100%). Following dehydration, samples were embedded in epoxy resin (812, DDSA, MNA, DMP30) and polymerized.

For semithin analysis, the sections (5–6 μm) were prepared using a Leica UC7 ultramicrotome, stained with methylene blue and azure II, and examined under a Carl Zeiss Axioscope 5 light microscope, AG, Oberkochen, Germany. For ultrastructural analysis, ultrathin sections (~60–70 nm) were prepared using a Leica UC7 ultramicrotome, Wetzlar, Germany mounted on copper grids, and, if necessary, contrasted with uranyl acetate and lead citrate. The sections were then examined using a JEM 1400Plus (Jeol) transmission electron microscope, Tokyo, Japan in bright-field mode at 80 kV.

### 2.5. Diabetes Mellitus Induction

Diabetes mellitus was induced in rats under inhalation anesthesia through intraperitoneal administration of streptozotocin (STZ) at a dose of 60 mg/kg body weight (in 0.1 M citrate buffer, pH 4.5). Prior to STZ administration, the rats were fasted overnight. After 2 days of STZ injection, blood glucose levels were measured using a glucometer. Animals with a blood glucose concentration >250 mg/dL (13.8 mmol/L) were considered to meet the criteria for experimental diabetes and were included in subsequent experimental procedures. A full-thickness excisional wound was created only after the diabetic phenotype had been confirmed [52,53].

### 2.6. Creation of Experimental Diabetic Wound Model

In experimental rats under inhalation anesthesia, a circular excision of a full-thickness skin flap, up to 10 mm in diameter, was performed on the pre-shaved interscapular area after surgical field preparation. The procedure was carried out using the DERMO PUNCH skin biopsy system (DMP) №10, with the wounds extending to the underlying fascia and skeletal muscle layers.

### 2.7. Treatment Design for Diabetic Wounds

The experiments were conducted on albino Wistar rats with mass of 250 ± 20 g (*n* = 30) with a model of diabetic wounds. The rats were randomly divided into five groups, with six rats in each group (*n* = 6):

- Two study groups received autologous RBCDCs loaded with either Ceftriaxone alone (RBCDCs (Ctx)) or EGF and Ceftriaxone (RBCDCs (EGF + Ctx));
- Two control groups received the same drugs in free form—either Ceftriaxone alone (FF(Ctx)) or EGF and Ceftriaxone (FF (EGF + Ctx));
- Another additional control group received standard topical application of the ointment “Levomekol” (Ctrl).

Each drug was injected into the wound using separate syringes at different points. In both the experimental and control groups, each rat received local wound infiltration through injections into the base and walls of the wound at several points, ensuring equal distribution of the infiltrated substance around the wound.

Wound care was performed daily, involving antiseptic treatment of the wound followed by the application of an aseptic dressing. The animals from the experimental groups were treated by the following technique: wound edges and bottom were injected with the sterile autologous RBCDCs containing either ceftriaxone alone or EGF and ceftriaxone. Additional injections were administered once daily at 24 and 48 h after the initial administration. Animals in the control groups were treated by injecting free-form drugs—either ceftriaxone alone or EGF and ceftriaxone—into the wound edges and bottom. Additional injections followed the same schedule as in the experimental groups. Animals in the control group (Ctrl) were treated with the standard method by the topical application of ointment “Levomecol” (combination of Chloramphenicol and Methyluracil). In the RBCDCs (EGF + Ctx) group, EGF and ceftriaxone were loaded into separate RBCDC preparations and were not co-encapsulated within the same carrier formulation. The separately prepared EGF-loaded and ceftriaxone-loaded RBCDCs were administered using different syringes at different points of the wound.

The study was designed primarily to compare RBCDC-mediated administration with the corresponding free-drug formulations containing the same active components. Unloaded RBCDCs, free EGF alone, and EGF-loaded RBCDCs without ceftriaxone were not included as separate experimental groups.

### 2.8. Assessment of the Therapeutic Efficacy of RBCDCs Containing EGF and Ceftriaxone in Diabetic Wound Healing

The therapeutic efficacy of RBCDCs containing EGF and ceftriaxone in diabetic wound healing was evaluated based on clinical indicators reflecting the progression of the inflammatory process. These included the time required for the disappearance of inflammatory infiltration around the wound, the time to normalization of cytological and histological profiles, and the completion of wound healing. In all experimental groups, wound area measurements were taken on days 3, 5, 7, and 9. Wound size was determined using a transparent film (palette) and millimeter paper. The average wound area (mm^2^) was calculated for each group of animals. To monitor the progression of wound healing in laboratory animals, clinical signs of healing were evaluated in addition to wound size. These included the rate of disappearance of local inflammatory symptoms such as hyperemia, edema, tissue infiltration, and pain. In addition, wounds were photographed on days 0, 3, 5, 7, and 9 to monitor changes in the wound area.

### 2.9. Cytological Assessment of Wound Healing Dynamics

To monitor the wound healing process, identify inflammatory phases, and evaluate the effectiveness of the applied treatment, the cytological profile of wound healing dynamics was studied in both experimental and control groups. For this purpose, during surgery and on the 3rd, 5th, 7th, and 9th days of treatment, smear samples were collected from the wound surface of the experimental animals. Both aspiration puncture biopsies from the wound and smear samples from the surface of the open wound were performed in the experimental and control groups. The cytological samples were fixed with 96% alcohol and stained with azure and eosin using the Romanovsky–Giemsa method. The samples were then examined under a Carl Zeiss Axioscope 5 light microscope.

### 2.10. Histological Assessment of Wound Healing Dynamics

For histological examination of wound tissues, animals are euthanized under isoflurane anesthesia on day 9 post-treatment and skin area with wound is cut out.

The harvested wound tissues were fixed in 10% neutral formalin. Tissue processing, including dehydration and paraffin impregnation, was carried out using the Leica HistoPearl Tissue Processor (Leica Biosystems, Nussloch, Germany). The fixed tissues were embedded in paraffin using the Tissue Embedding Center BK-TEII (BIOBASE, Jinan, China) and sectioned into serial slices via microtomy using the Automatic Microtome (BIOBASE). The tissue sections were then stained with hematoxylin and eosin (H&E) using the Tissue Stainer BK-TS2 (BIOBASE).

Following staining, the tissue samples were examined microscopically and photographed using a micro camera.

### 2.11. Quantification of Collagen Deposition and Angiogenesis in the Wound Tissue

Collagen deposition in the wound tissue was evaluated using Masson’s trichrome staining. Briefly, paraffin-embedded wound tissue sections were stained with Masson’s trichrome according to the manufacturer’s protocol. Collagen fibers were stained blue, whereas muscle fibers and cytoplasm were stained red, allowing visualization and assessment of collagen deposition within the wound tissue. The extent of collagen deposition within the wound bed was assessed by light microscopy. Angiogenesis was evaluated histologically by assessing the degree of neovascularization within the granulation tissue. The extent of angiogenesis was scored using a semi-quantitative grading system and expressed as a percentage of the maximum score.

### 2.12. Analysis of Growth Factor Concentrations in Wound Tissue

The harvested wound tissues were collected on day 9 post-treatment, as described in Section 2.10, and stored at −80 °C for subsequent analysis. The harvested tissues were homogenized and combined with 0.1 mL of lysis buffer. According to the manufacturer’s instructions, standards and pretreated samples were then pipetted into the wells of a 96-well ELISA plate. Growth factor concentrations were measured using an ELISA reader (Cyntation 5 device) and rat-specific ELISA kits for EGF (Cloud-Clone Corp, Wuhan, China) in accordance with the manufacturers’ protocols.

### 2.13. Statistical Analysis

Statistical analyses were performed using GraphPad Prism version 10.4.2 (GraphPad Software Inc., San Diego, CA, USA). Quantitative data are presented as mean ± SD or mean ± SEM, as specified in the corresponding tables and figure legends. To evaluate the effects of treatment and time on wound healing percentage, a two-way repeated-measures ANOVA was conducted, followed by Dunnett’s multiple comparisons test. Measurements from the same animals were matched across time points, and no missing values were present in the longitudinal wound-healing dataset. Statistical significance was defined as *p* < 0.05. Only statistically significant differences are indicated on the graphs, represented by asterisks (*, **, ***, ****). In addition, hash symbols (#, ##, ###) were used to indicate statistically significant differences between the free and encapsulated formulations, which were calculated separately using unpaired two-tailed *t*-tests.

## 3. Results

### 3.1. Characterization of Drug-Loaded RBCDCs

#### 3.1.1. Ceftriaxone Encapsulation Efficiency

The encapsulation efficiency of ceftriaxone in RBCDCs was determined by HPLC analysis of the lysed carrier fraction. After accounting for the dilution factors, the mean ceftriaxone concentration in the RBCDC suspension was approximately 77.6 mg/mL, corresponding to an encapsulated amount of approximately 605.1 mg in 7.8 mL of the final RBCDC suspension. Based on an initial ceftriaxone amount of 1000 mg, the calculated encapsulation efficiency was approximately 60.5%.

#### 3.1.2. In Vitro Ceftriaxone Release

The in vitro release profile of ceftriaxone from RBCDCs was evaluated over 24 h using a membrane dialysis method. Cumulative ceftriaxone release increased rapidly during the initial phase, reaching 21.64% at 0.25 h, 36.97% at 0.5 h, 49.61% at 1 h, and 66.34% at 3 h. Thereafter, the release rate markedly decreased and approached a plateau, with cumulative release reaching 71.02% at 17 h and 71.14% at 24 h. Thus, approximately two-thirds of ceftriaxone was released during the first 3 h, followed by a substantially slower release phase up to 24 h [51].

#### 3.1.3. In Vitro Release Profile of EGF from EGF-Loaded RBCDCs 

The in vitro release of EGF from EGF-loaded RBCDCs was evaluated using a dialysis membrane system in six independently analyzed preparations. EGF was quantifiable in the external dialysis medium in all six EGF-loaded RBCDC at 15 min, 30 min, and 1 h, with mean concentrations of 161.6 ± 71.7 pg/mL, 137.6 ± 58.9 pg/mL, and 96.0 ± 49.3 pg/mL, respectively. At 1.5 h, EGF remained quantifiable with a mean concentration of 79.9 ± 43.9 pg/mL. At 2 h and 3 h, EGF remained quantifiable with mean concentrations of 78.0 ± 16.9 pg/mL and 47.7 ± 10.5 pg/mL, respectively. Following completion of the dialysis experiment, EGF remained detectable in the intramembrane RBCDC-containing fraction in all six preparations, with a mean concentration of 171.4 ± 47.1 pg/mL.

#### 3.1.4. Physicochemical Characteristics of EGF-Loaded RBCDCs: Particle Size and Zeta Potential 

Nanoparticle tracking analysis demonstrated a submicron particle population. Across four independently analyzed preparations, the median particle diameter (X50) was 194.4 ± 6.0 nm, while the mean particle diameter was 228.6 ± 10.6 nm. The average X10 and X90 values were approximately 118.8 nm and 335.2 nm, respectively, with a mean distribution span of approximately 1.10. The mean zeta potential was −6.65 ± 4.33 mV.

### 3.2. Microscopic Examination of Drug-Loaded RBCDCs

**Figure 1:**
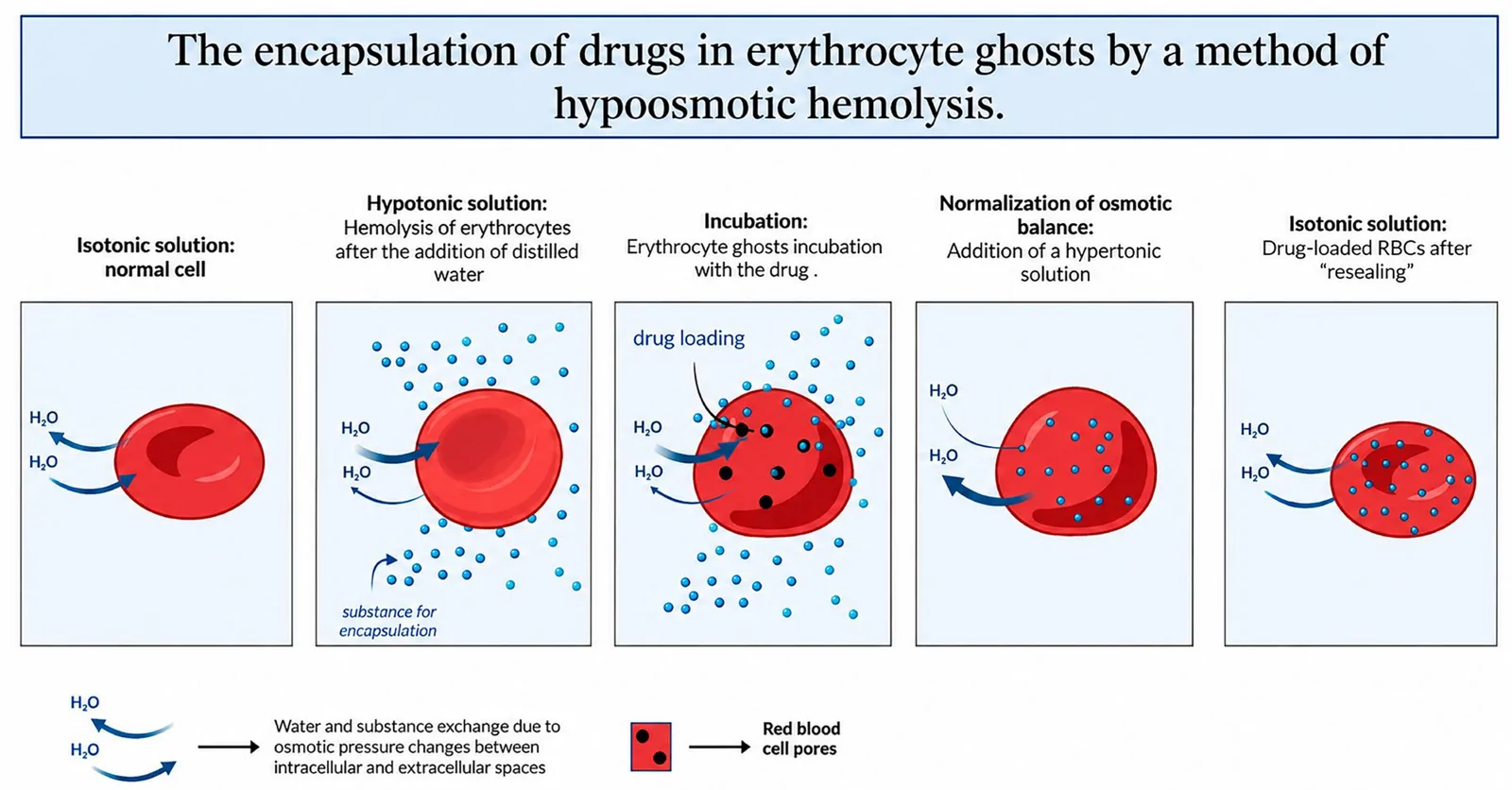
The encapsulation of the drug in erythrocyte ghosts by the method of hypoosmotic hemolysis. Schematic of the preparation process of pharmacocytes—drug-loaded RBC-derived carriers using hypoosmotic hemolysis.

Drug loaded RBCDCs have been prepared by the method of hypoosmotic hemolysis according to the protocol as described in the section Methods. The main stages of the preparation process of pharmacocytes based on hypoosmotic hemolysis method are shown in Figure 1.

**Figure 2:**
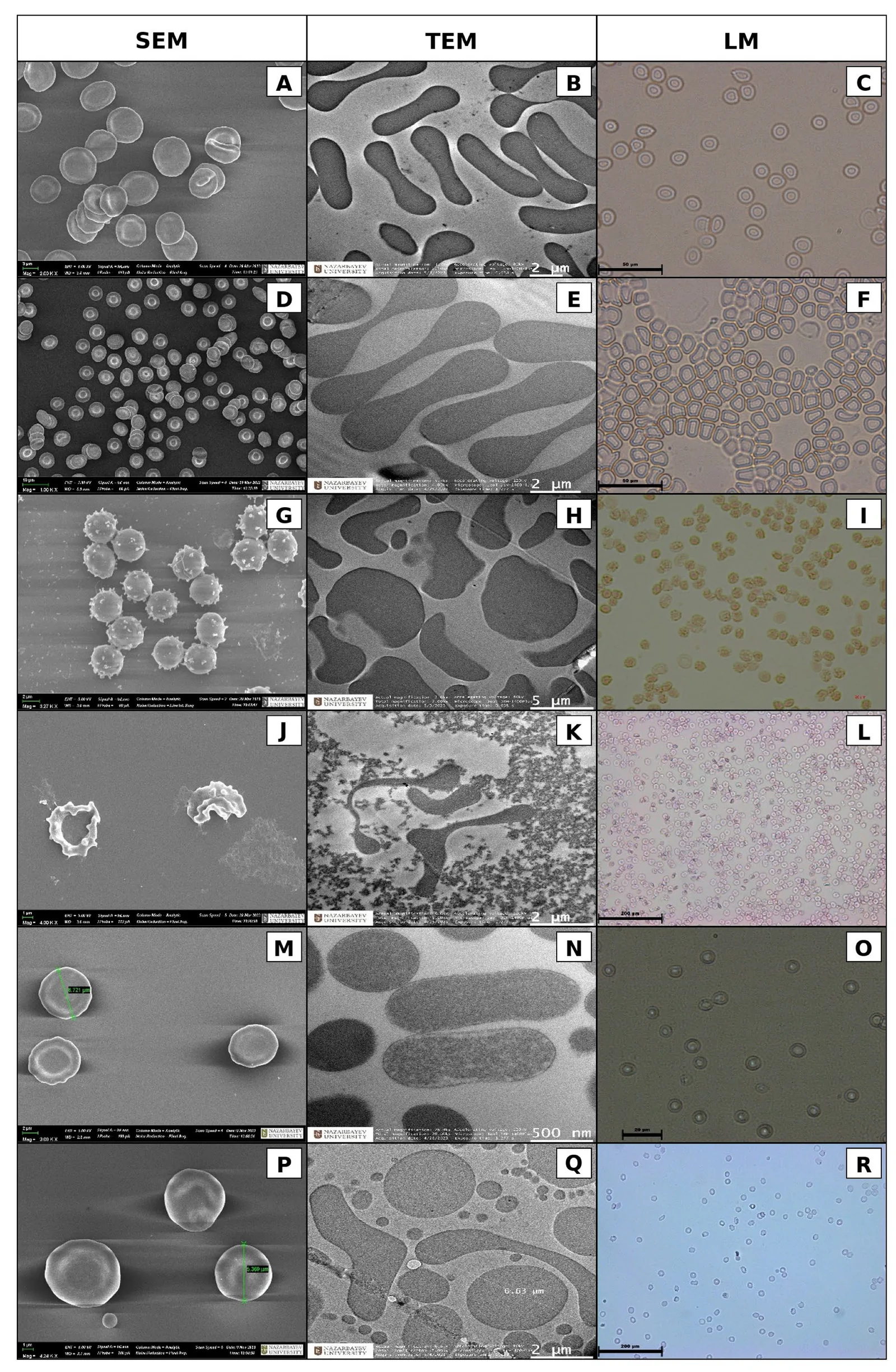
Morphostructural changes in erythrocytes during drug encapsulation using method of hypoosmotic hemolysis. Stages of preparation of drug-loaded RBCs: (**A**–**C**)—RBCs after plasma removal; (**D**–**F**)—RBCs after three times washing; (**G**–**I**)—hemolysis of RBCs after addition of distilled water; (**J**–**L**)—RBC ghosts after centrifugation; (**M**–**O**)—RBCDCs after incubation with the drug; (**P**–**R**)—drug loaded RBCDCs after “resealing”. Scanning electron microscope (SEM) images (**A**,**D**,**G**,**J**,**M**,**P**) were obtained using a Auriga Crossbeam 540 (Carl Zeiss) with an InLens detector at 2000×, 1000×, 3000×, 4000×, 3000×, and 4000× magnifications, respectively. Transmission electron microscope (TEM) images (**B**,**E**,**H**,**K**,**N**,**Q**) were obtained using a JEM 1400Plus (Jeol) in bright-field mode at 2500×, 4000×, 2000×, 5000×, 2000×, and 5000× magnifications, respectively. The light microscopy (LM) images (**C**,**F**,**I**,**L**,**O**,**R**) were obtained using a Leica DM 4000 light microscope at 40× magnification. The magnification for each panel is specified individually in the figure legend, and scale bars are shown in each panel.

Microscopic examination of erythrocytes was performed at each stage of drug loading to identify morphological changes. The key stages of drug encapsulation into erythrocytes via hypoosmotic hemolysis were visualized using scanning electron microscopy (SEM), transmission electron microscopy (TEM), and light microscopy (LM). Microscopic alterations in the morphofunctional and structural characteristics of drug-loaded RBCs induced by hypoosmotic hemolysis are shown in Figure 2.

Figure 2 illustrates the data obtained from the microscopic analysis of morphological changes in RBCs at each stage of the drug incorporation process. The experimental results revealed a positive correlation between erythrocyte morphology changes and variations in the osmotic pressure of the incubation solution. These findings highlight the dynamic changes occurring during RBCDC preparation and underscore the specific morphofunctional and structural alterations associated with drug loading by the hypoosmotic hemolysis method.

### 3.3. Glucose Measurement

Blood glucose monitoring revealed persistently elevated glycemia in the experimental animals following streptozotocin injection, with an average blood glucose level of 27.5 mmol/L on day 3. Additionally, the rats exhibited glucosuria, polydipsia, and polyphagia.

### 3.4. Therapeutic Efficacy of RBCDCs Containing EGF and Ceftriaxone on Diabetic Wound Healing

**Figure 3:**
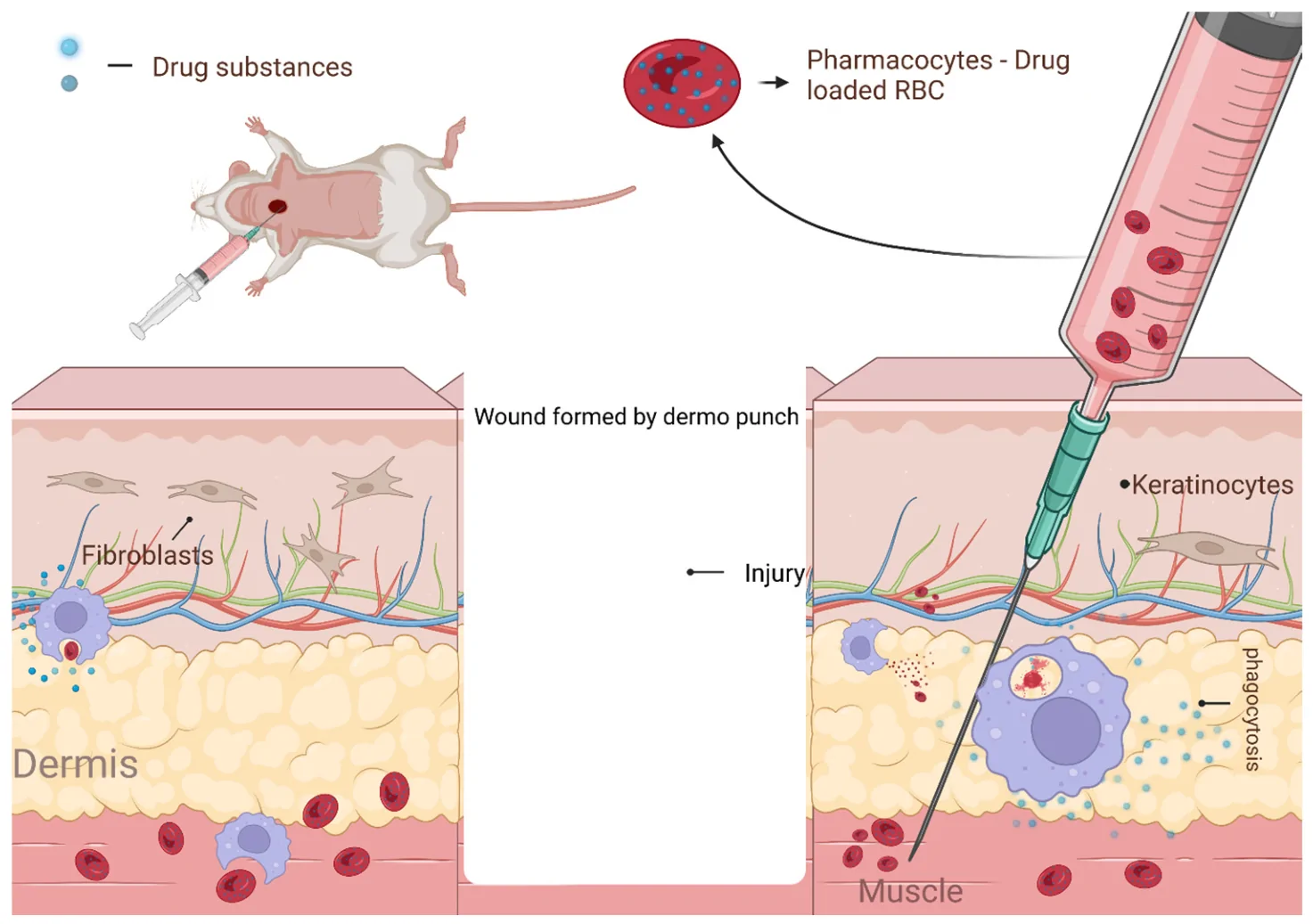
Schematic illustration of the treatment design for diabetic wound healing using the topical injection of drug-loaded RBCDCs.

We developed drug-loaded RBCDCs for targeted delivery of growth factors and antibiotics to accelerate diabetic wound healing. The animals from the experimental groups were treated by the developed technique: wound edges and bottom were injected with the sterile autologous RBCDCs containing either ceftriaxone alone or EGF and ceftriaxone. Additional injections were administered once daily at 24 and 48 h after the initial administration, as schematically shown in Figure 3.

Two study groups received autologous RBCDCs loaded with either Ceftriaxone alone (RBCDCs (Ctx)) or EGF and Ceftriaxone (RBCDCs (EGF + Ctx)). Two control groups received the same drugs in free form—either Ceftriaxone alone (FF(Ctx)) or EGF and Ceftriaxone (FF (EGF + Ctx)). Another additional control group received standard topical application of the ointment “Levomekol” (Ctrl).

**Figure 4:**
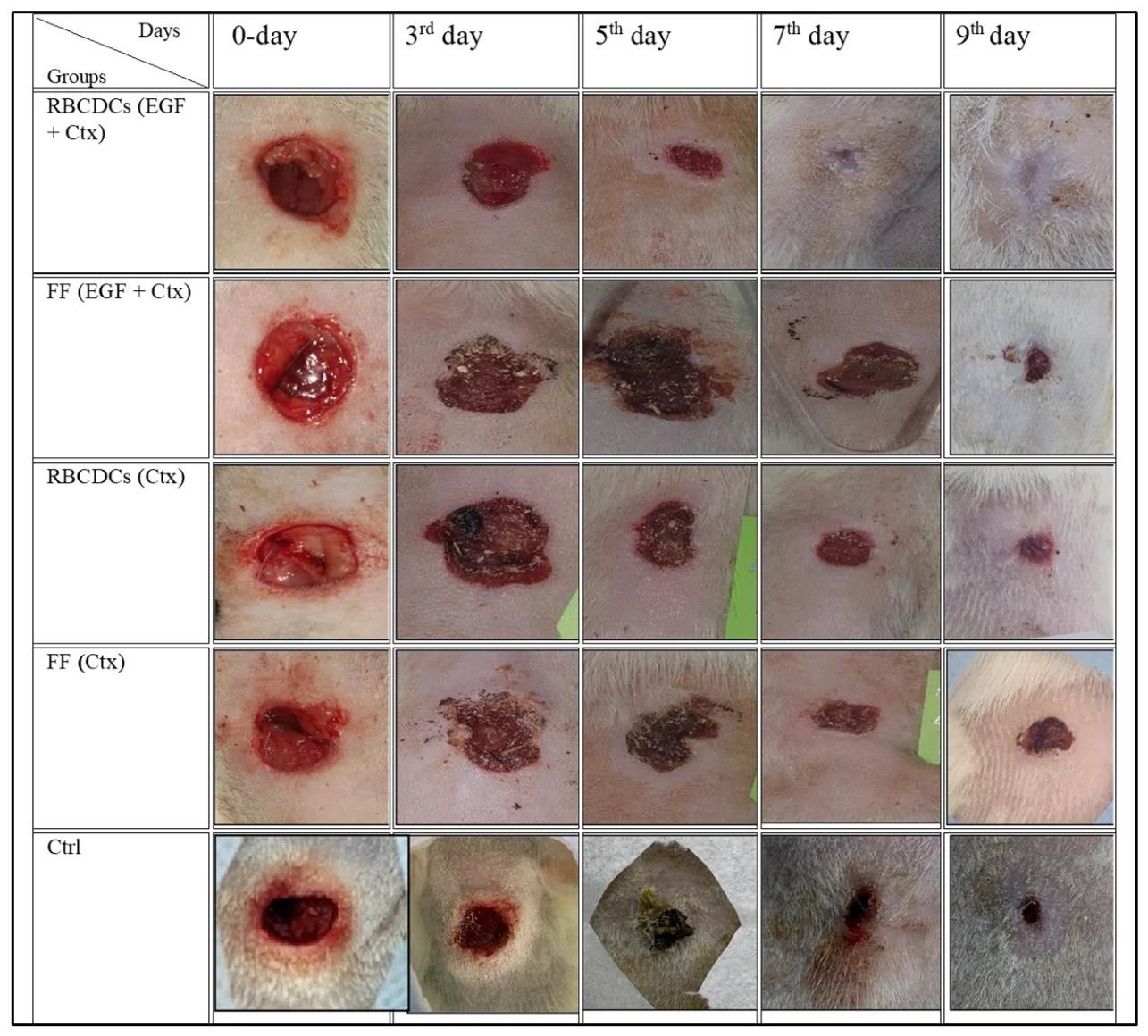
Treatment of wounds with drug-loaded RBCDCs containing ceftriaxone and EGF accelerated the regenerative process and promoted early maturation of granulation tissue. Images showing wound closure progression in the following five groups on days 0, 3, 5, 7, and 9: RBCDCs loaded with EGF and ceftriaxone (RBCDCs (EGF + Ctx)); free EGF and ceftriaxone (FF (EGF + Ctx)); RBCDCs loaded with ceftriaxone alone (RBCDCs (Ctx)); free ceftriaxone alone (FF (Ctx)); and control (Ctrl).

The therapeutic efficacy of RBCDCs loaded with EGF and ceftriaxone was demonstrated by significantly accelerated diabetic wound healing compared with the control groups, as shown in Figure 4.

The experimental groups demonstrated earlier and more effective wound healing than the control groups. By day 3 after treatment initiation, only mild local inflammatory signs remained in the experimental groups, including slight skin hyperemia, minimal edema, and moderate soft tissue infiltration around the wound. By day 5, local inflammatory signs had completely resolved in the experimental groups, accompanied by a significant reduction in wound size compared with the control groups. Skin hyperemia, edema, and soft tissue infiltration were absent in the experimental groups, while only reduced in the control groups.

By day 7, no signs of local inflammation were observed in the experimental groups. Wounds had markedly contracted, healed by secondary intention, and were covered with scar tissue and fully healed (100.0 ± 0.0%). In contrast, although wound contraction and granulation tissue formation were also observed in the control groups, the healing process remained slower and less advanced.

By day 9, treatment with RBCDCs loaded with EGF and ceftriaxone continued to demonstrate complete wound closure, indicating superior therapeutic efficacy compared with both the control group and the corresponding free-drug formulations. Overall, topical administration of drug-loaded RBCDCs significantly accelerated wound repair by promoting early resolution of inflammation, enhanced granulation tissue maturation, and faster tissue regeneration.

### 3.5. Assessment of the Effect of Drug-Loaded RBCDCs on Wound-Healing Dynamics

**Figure 5:**
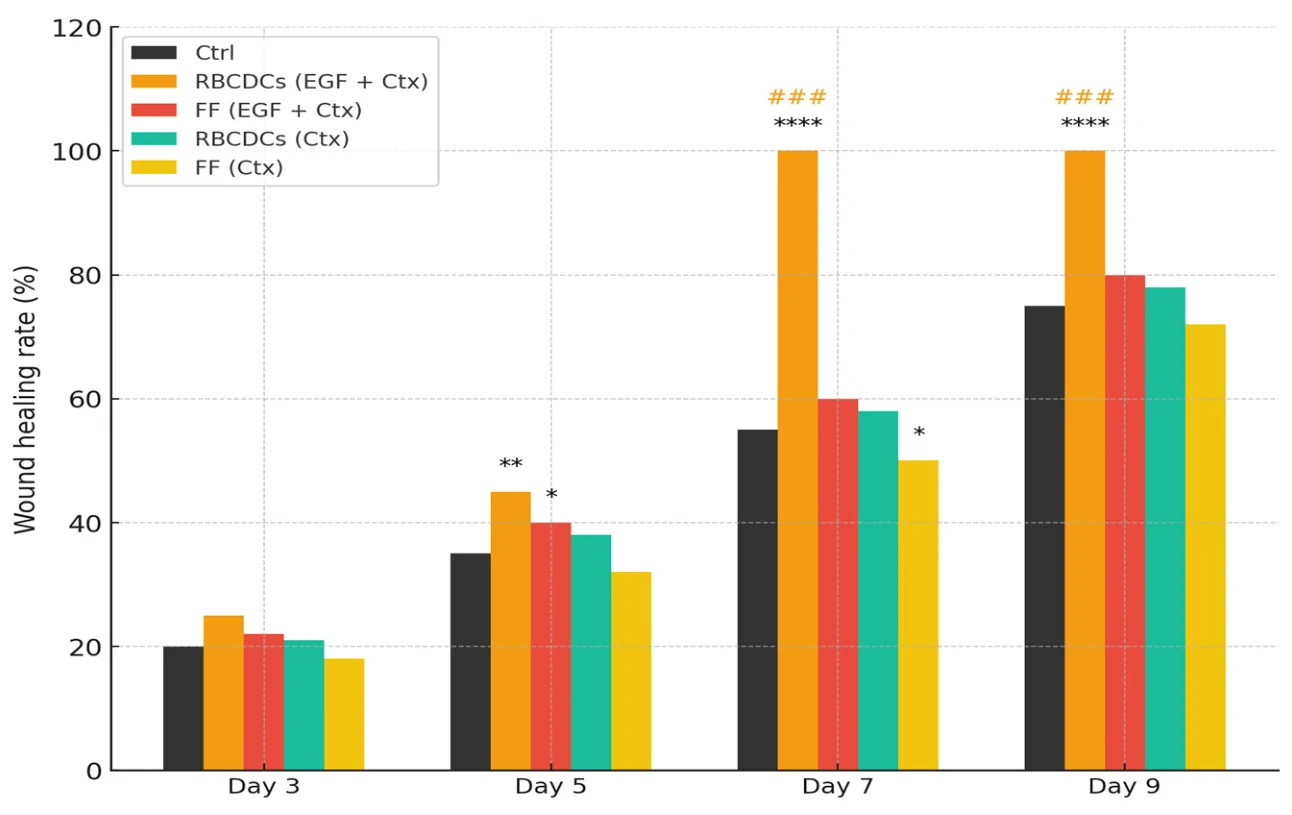
Wound-healing dynamics in the treatment groups. Percentage wound closure is shown on Days 3, 5, 7, and 9 for Ctrl, RBCDCs (EGF + Ctx), FF (EGF + Ctx), RBCDCs (Ctx), and FF (Ctx) (*n* = 6 per group). The graph shows group mean values; corresponding mean ± SEM values are provided in Table 1. Longitudinal wound-healing data were analyzed using two-way repeated-measures ANOVA followed by Dunnett’s multiple comparisons test. Asterisks indicate statistically significant differences compared with the Ctrl group (* *p* < 0.05; ** *p* < 0.01; **** *p* < 0.0001). Hash symbols indicate comparisons between RBCDC formulations and their corresponding free-drug formulations (### *p* < 0.001).

Macroscopic wound-healing progression was evaluated by measuring wound area on Days 0, 3, 5, 7, and 9 after treatment initiation. Wound healing was expressed as the percentage reduction in wound area relative to the initial wound size. Quantitative data are summarized in Table 1 and Figure 5 and are presented as mean ± standard error of the mean (SEM) for each treatment group (*n* = 6).

The RBCDCs (EGF + Ctx) group demonstrated the most rapid wound healing. By day 7, complete wound healing (100.0 ± 0.0%) was observed in this group, whereas only partial healing was observed in all other groups. Complete healing in the RBCDCs (EGF + Ctx) group was maintained through day 9.

The wound healing dynamics in each treatment group of rats (mean ± SD) are shown in Figure 5.

Treatment with RBCDCs loaded with EGF and ceftriaxone resulted in the greatest acceleration of wound healing throughout the observation period. Compared with the control group and the corresponding free-drug formulations, the RBCDC (EGF + Ctx) group exhibited significantly faster wound closure, particularly on Days 5, 7, and 9. RBCDCs loaded with ceftriaxone alone also promoted wound healing compared with free ceftriaxone, although the therapeutic effect was less pronounced than that observed with the combined EGF and ceftriaxone formulation. These findings demonstrate improved wound-healing outcomes in the RBCDC-treated groups compared with the corresponding free-drug formulations, with the most pronounced effect observed in the RBCDCs (EGF + Ctx) group.

### 3.6. Cytological Evaluation of Wound Healing

**Figure 6:**
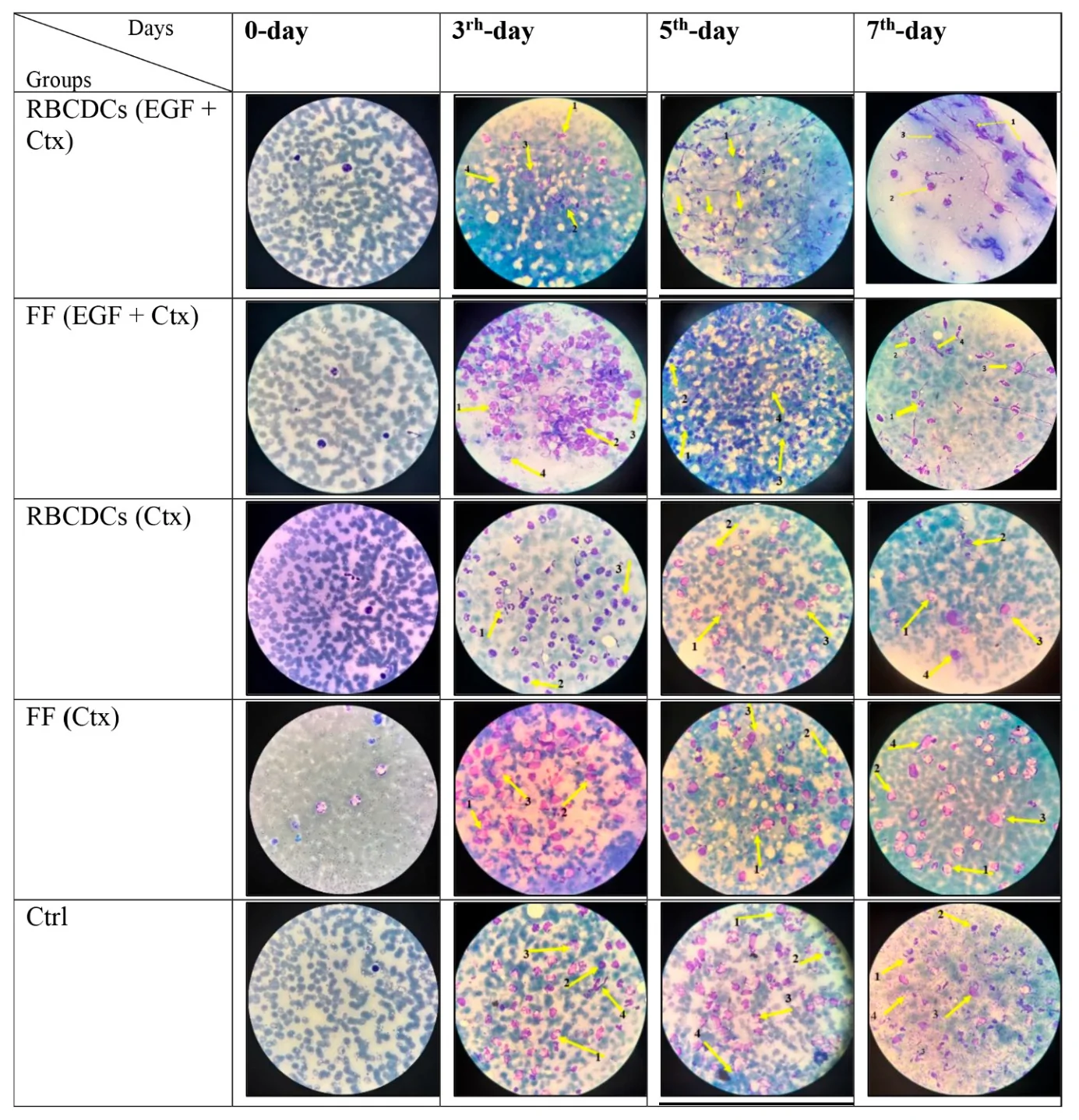
Comparative cytological profiles of wound healing in the experimental and control groups. Azure–eosin staining according to Romanowsky–Giemsa, ×1000 magnification. Representative cytological smears obtained on days 0, 3, 5, and 7 from wounds treated with RBC-derived carriers (RBCDC) loaded with EGF and ceftriaxone (RBCDC (EGF + Ctx)); free EGF and ceftriaxone (FF (EGF + Ctx)); RBCDC loaded only with ceftriaxone (RBCDC (Ctx)); free ceftriaxone (FF (Ctx)); and control group wounds (Ctrl). The images allow for a comparison of the dynamics of inflammatory and reparative cellular responses during the wound healing process.

**Figure 7:**
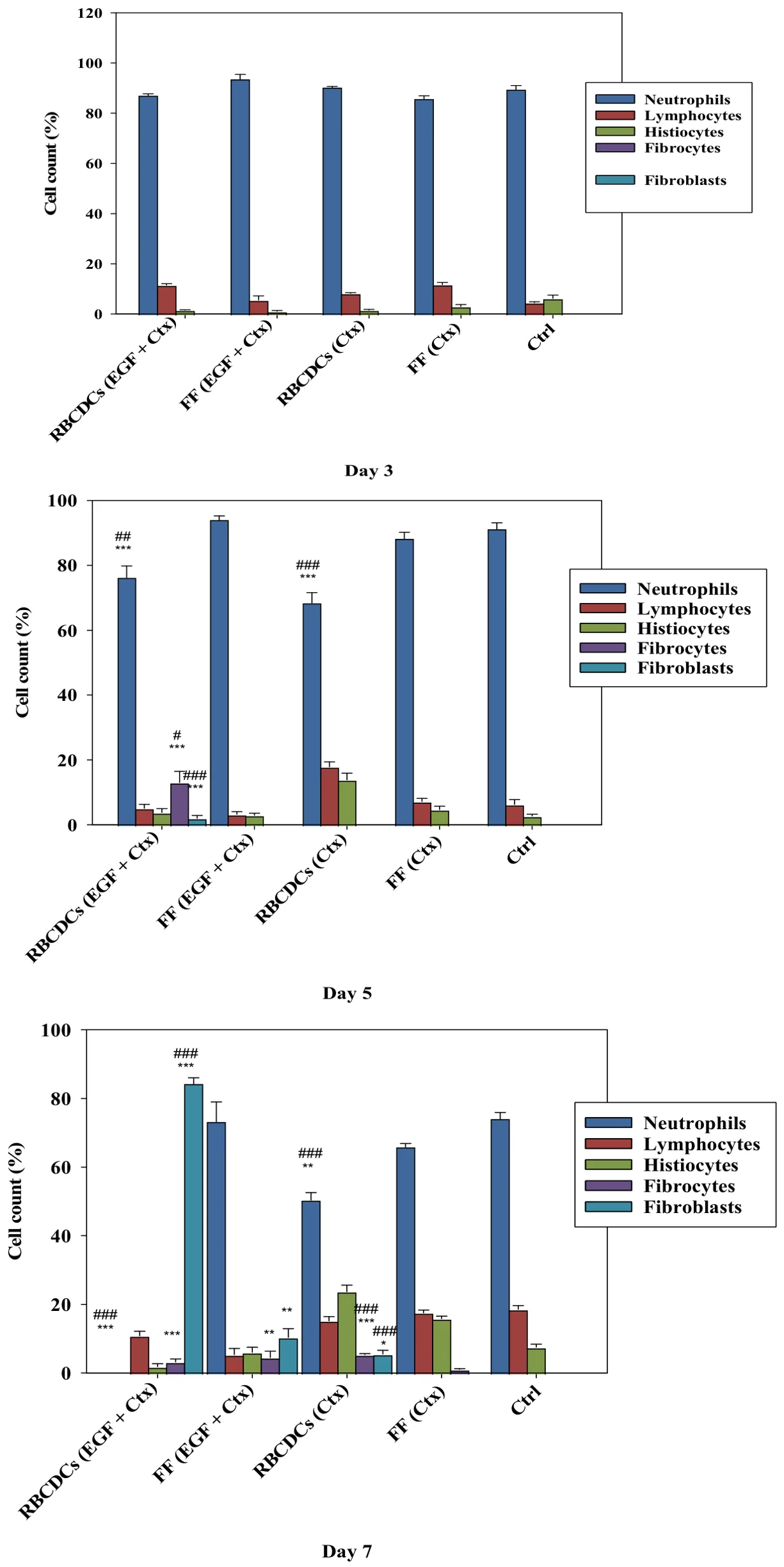
Quantitative analysis of inflammatory and reparative cell populations in wound tissue on days 3, 5, and 7 after treatment (*n* = 6). Cell counts were determined manually using a Carl Zeiss Axioscope 5 light microscope. Data are presented as mean ± SD. At each time point, statistical analysis was performed using one-way ANOVA followed by Dunnett’s post hoc test to compare experimental groups with the Ctrl group. Comparisons between RBCDC formulations and their corresponding free-drug (FF) groups were performed using a two-tailed Welch’s *t*-test. Statistical significance was defined as *p* < 0.05. Asterisks indicate comparisons with the control group (* *p* < 0.05, ** *p* < 0.01, *** *p* < 0.001), whereas hash symbols indicate comparisons between RBCDC formulations and their corresponding free-drug formulations (# *p* < 0.05, ## *p* < 0.01, ### *p* < 0.001).

Cytological examination was performed to monitor wound healing progression, determine the stage of the wound healing process, and evaluate the therapeutic efficacy of targeted drug delivery. Representative cytological images of all treatment groups are shown in Figure 6, while quantitative analysis of inflammatory and reparative cell populations is presented in Figure 7.

On Day 0, cytological smears from all groups contained only peripheral blood cells without evidence of inflammatory infiltration. By Day 3, all groups exhibited inflammatory cell infiltration characterized predominantly by neutrophils, confirming the onset of the inflammatory phase of wound healing. However, the severity of inflammation differed substantially among treatment groups. The RBCDC (EGF + Ctx) group demonstrated only mild neutrophilic infiltration accompanied by lymphocytes and histiocytes, whereas the free-drug groups and the untreated controls showed pronounced neutrophilic infiltration with numerous degenerating neutrophils, active phagocytosis, and abundant bacterial flora. The RBCDC (Ctx) group also exhibited inflammatory infiltration, although it was less pronounced than in the corresponding free-drug group.

By Day 5, the RBCDC (EGF + Ctx) group exhibited a clear transition from the inflammatory to the proliferative phase of healing. Cytological smears revealed only mild inflammatory infiltration together with fibrin strands, fibroblasts, fibrocytes, histiocytes, and lymphocytes, indicating active granulation tissue formation. In contrast, moderate to severe inflammation persisted in the free-drug groups and controls, where neutrophils remained the predominant cell population and bacterial contamination was still observed in some specimens.

By Day 7, wounds treated with RBCDC (EGF + Ctx) showed advanced tissue regeneration characterized by complete resolution of inflammatory infiltration, abundant fibroblasts and fibrin fibers, and early maturation of granulation tissue. The RBCDC (Ctx) group also demonstrated enhanced reparative activity, although to a lesser extent. In comparison, the free-drug formulations and untreated controls continued to exhibit residual neutrophilic infiltration with less developed reparative changes, indicating delayed wound healing.

Quantitative cytological analysis confirmed these qualitative observations (Figure 7).

The differences in the numbers of inflammatory and reparative cells between treatment groups on days 3, 5, and 7 post-treatment are shown in Figure 7.

On Day 3, neutrophil counts did not differ significantly between the RBCDC formulations, their corresponding free-drug groups, or the untreated control group, indicating that inflammatory cell recruitment was comparable during the early stage of healing. By Day 5, neutrophil numbers were significantly reduced in the RBCDC (EGF + Ctx) and RBCDC (Ctx) groups compared with the control group (both ** *p* < 0.001). In addition, all RBCDC formulations exhibited significantly lower neutrophil counts than their corresponding free-drug formulations, demonstrating a superior anti-inflammatory effect of erythrocyte-mediated drug delivery.

By Day 7, neutrophilic infiltration remained significantly lower in the RBCDC (EGF + Ctx) and RBCDC (Ctx) groups than in both the untreated control and the corresponding free-drug groups, indicating more rapid resolution of inflammation.

This reduction was accompanied by a marked increase in reparative cell populations. On Day 5, fibrocyte numbers were significantly higher in the RBCDC (EGF + Ctx) group than in the control group (** *p* < 0.001), reflecting accelerated granulation tissue formation. By Day 7, all RBCDC formulations exhibited significantly greater fibroblast numbers than their corresponding free-drug formulations, consistent with enhanced extracellular matrix synthesis and tissue remodeling.

Overall, cytological evaluation demonstrated that erythrocyte-mediated delivery of growth factors and ceftriaxone accelerated the transition from the inflammatory to the proliferative phase of diabetic wound healing. Among all treatment groups, RBCDCs loaded with EGF and ceftriaxone produced the most pronounced anti-inflammatory and regenerative responses, leading to earlier granulation tissue maturation and more advanced wound repair than the corresponding free-drug formulations.

**Figure 8:**
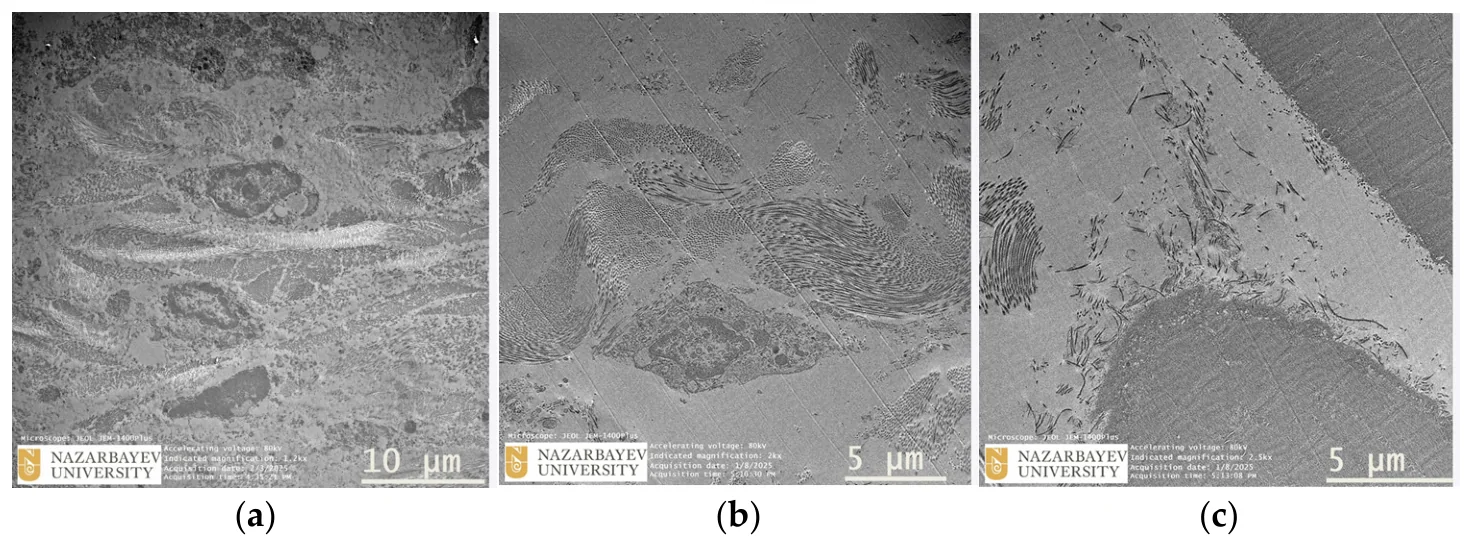
Representative scanning electron microscopy (SEM) images of wound healing in the RBCDC (EGF + Ctx) group. On Day 5 after treatment initiation, SEM demonstrated early granulation tissue formation (**a**) and numerous activated fibroblasts producing abundant fibrin fibers (**b**,**c**), indicating accelerated tissue repair.

The wound healing process was further evaluated by scanning electron microscopy (SEM) (Figure 8). In the RBCDC (EGF + Ctx) group, SEM revealed early granulation tissue formation by Day 5 after treatment initiation (Figure 8a), accompanied by numerous activated fibroblasts producing abundant fibrin fibers (Figure 8b,c). In contrast, active granulation tissue formation in the remaining treatment groups was not observed until Day 6 or later, indicating delayed progression to the proliferative phase of wound healing.

Overall, comparative cytological and ultrastructural analyses demonstrated marked differences in wound healing among the treatment groups. The RBCDC (EGF + Ctx) group exhibited the most advanced regenerative response, characterized by earlier resolution of inflammation, accelerated granulation tissue formation, and enhanced fibroblast activation compared with both the RBCDC (Ctx), FF (Ctx), and untreated control groups. In contrast, wounds treated with ceftriaxone alone, either encapsulated in RBCDCs or administered in free form, exhibited delayed and less extensive regenerative changes. In these groups, fibroblasts first appeared on Day 7 and were present in relatively low numbers, indicating reduced fibroblast activation and slower tissue remodeling.

### 3.7. Histological Characteristics of Wound Healing 

**Figure 9:**
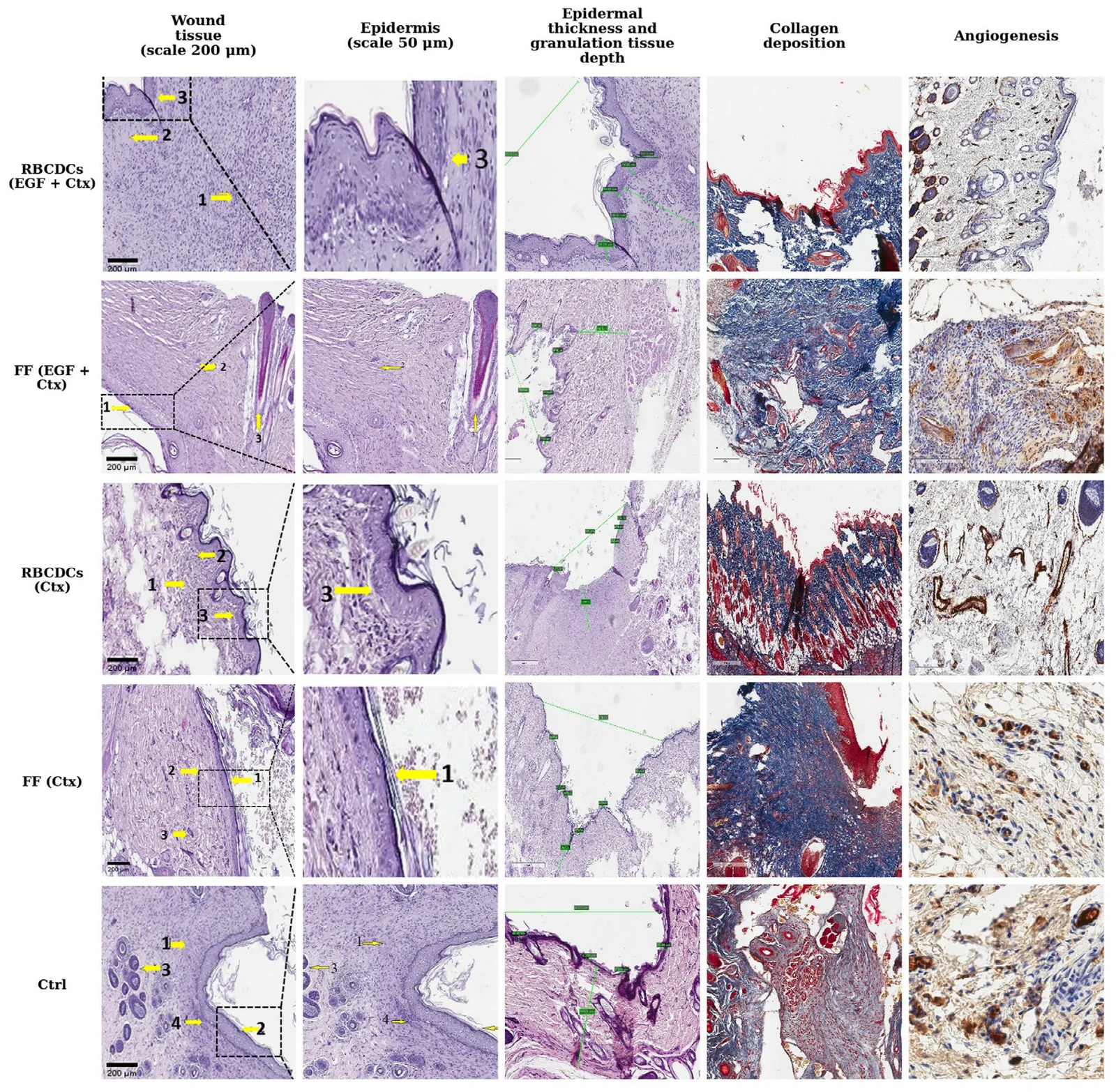
Representative histological images of wound healing in the experimental groups on day 9 after surgery. The figure is organized into five columns. Column 1 (Wound tissue) shows the overall wound area and surrounding tissue (H&E staining; scale bar = 200 μm). Column 2 (Epidermis) presents enlarged views of the boxed regions shown in Column 1, illustrating epidermal regeneration (H&E staining; scale bar = 50 μm). Column 3 (Epidermal thickness and granulation tissue depth) shows the corresponding histological sections used for assessment of epidermal thickness and granulation tissue depth. Column 4 (Collagen deposition) shows collagen deposition assessed by Masson’s trichrome staining (×40 magnification); collagen fibers are stained blue. Column 5 (Angiogenesis) shows the immunohistochemical assessment of angiogenesis using [insert marker, e.g., CD31] (×40 magnification). The arrows and numerical labels in Columns 1–3 indicate the respective histological structures or measurement regions described in the Results and Methods sections. Experimental groups: RBCDCs (EGF + Ctx), FF (EGF + Ctx), RBCDCs (Ctx), FF (Ctx), and Ctrl (Levomekol-treated control group).

The histological assessment of skin wounds of rats in different treatment groups on the 9th day after surgery is shown in Figure 9. Histological examination was performed to evaluate the degree of re-epithelialization, granulation tissue formation, collagen deposition, and angiogenesis during wound healing.

Histological evaluation demonstrated distinct differences in tissue regeneration among the experimental groups. Treatment with RBCDCs loaded with growth factors and ceftriaxone markedly accelerated wound repair compared with both the control and corresponding free-drug formulations. The RBCDCs (EGF + Ctx) group exhibited the most advanced stage of healing, characterized by a markedly thickened and continuous epidermis with a smooth surface, indicating nearly complete re-epithelialization. The dermis consisted predominantly of mature fibrous connective tissue with only scattered lymphocytic infiltration, suggesting resolution of inflammation and progression toward tissue remodeling. Skin appendages were not yet regenerated. The FF (EGF + Ctx) group also demonstrated complete epidermal coverage; however, the regenerated epidermis was thinner than that observed in the corresponding RBCDCs-treated group. The dermis contained mature hypocellular connective tissue with minimal inflammatory cell infiltration, and preserved skin appendages were detected only in adjacent healthy tissue.

In the RBCDCs (Ctx) group, young granulation tissue was observed beneath the epidermis together with mild inflammatory infiltration. Moderate fibrosis remained within the hypodermis, indicating that tissue remodeling was still ongoing. Similar findings were observed in the FF (Ctx) group, although epidermal thinning and more pronounced inflammatory infiltration were evident. The control (Ctrl) group exhibited delayed wound healing characterized by a flattened epidermis, persistent inflammatory cell infiltration within the dermis and hypodermis, and extensive fibrosis surrounding the wound area, indicating incomplete tissue regeneration.

Overall, histological findings demonstrated that erythrocyte-based delivery of growth factors combined with ceftriaxone substantially enhanced re-epithelialization, accelerated granulation tissue maturation, reduced inflammatory infiltration, and promoted tissue remodeling compared with the corresponding free-drug formulations.

### 3.8. Quantitative Assessment of Epidermal Thickness

Re-epithelialization represents a critical stage of cutaneous wound repair, restoring the epidermal barrier and protecting the wound from microbial invasion and excessive fluid loss. Increased epidermal thickness is widely recognized as a quantitative histological marker of successful re-epithelialization [54]. Therefore, epidermal thickness was measured on day 9 to quantitatively assess the regenerative response in each treatment group.

**Figure 10:**
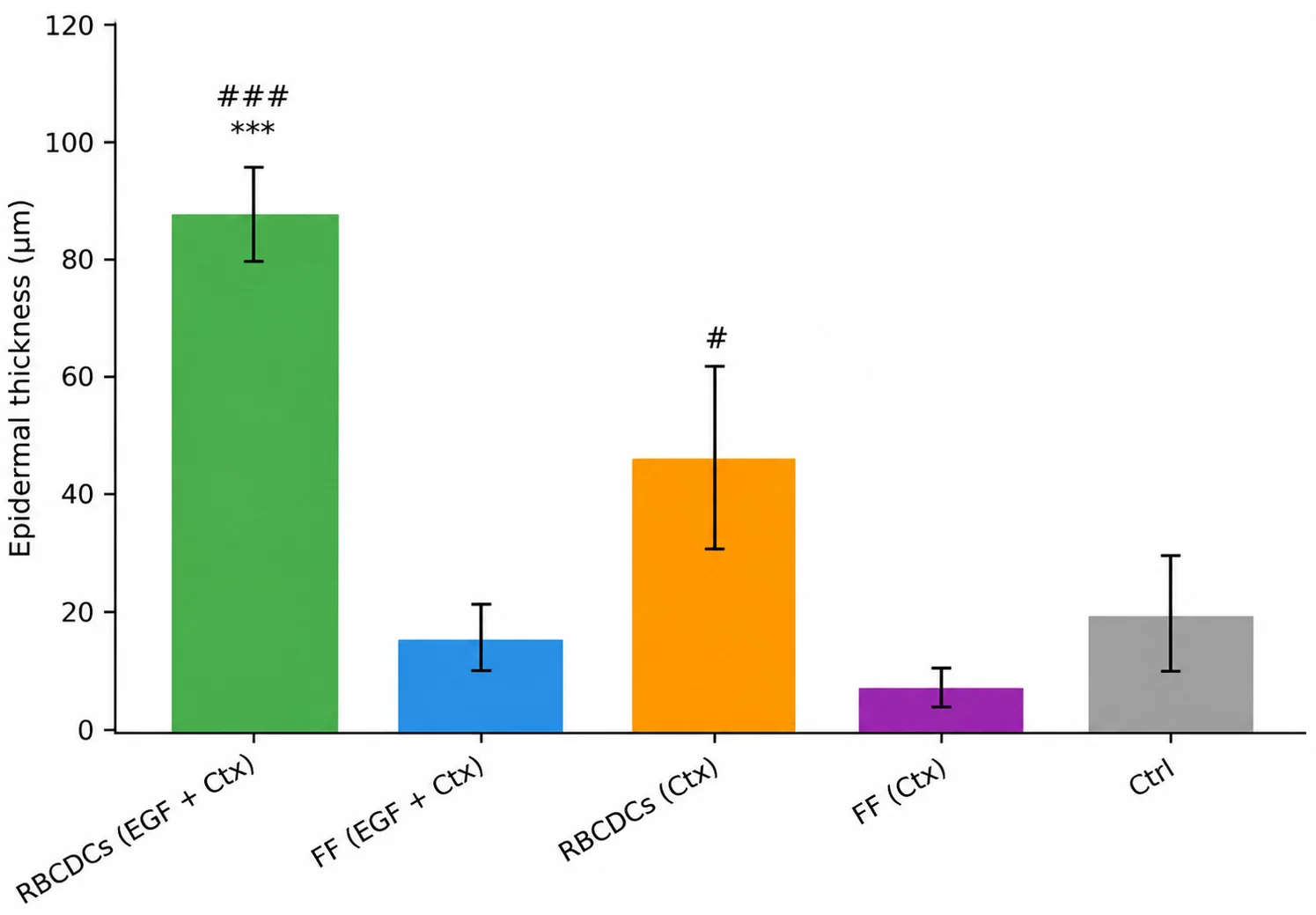
Quantitative assessment of epidermal thickness on day 9 after wound induction. Epidermal thickness was measured in histological sections from all experimental groups. Data are presented as the mean ± SEM. Statistical analysis was performed using one-way ANOVA followed by Dunnett’s post hoc test to compare each experimental group with the control (Ctrl) and Tukey’s multiple comparisons test to compare RBC-derived carrier formulations (RBCDCs) with their corresponding free-drug formulations (FF). Statistical significance compared with the control group is indicated as *** *p* < 0.001. Statistical significance between RBCDC formulations and their corresponding free-drug formulations is indicated as # *p* < 0.05, and ### *p* < 0.001.

The quantitative analysis of epidermal thickness is presented in Figure 10. The RBCDCs (EGF + Ctx) group exhibited the greatest epidermal thickness (88.40 ± 8.08 μm), significantly exceeding both the Ctrl group (*** *p* < 0.001) and the corresponding FF (EGF + Ctx) group (### *p* < 0.001). The RBCDCs (Ctx) group (46.12 ± 14.65 μm) showed significantly greater epidermal thickness than the corresponding FF (Ctx) group (# *p* < 0.05), whereas neither group differed significantly from the Ctrl group. The FF (EGF + Ctx) (16.41 ± 4.29 μm) and FF (Ctx) (7.66 ± 1.33 μm) groups did not differ significantly from the control (ns).

Quantitative morphometric analysis confirmed the histological observations (Figure 10). On day 9 after wound induction, epidermal thickness was quantitatively evaluated in all experimental groups (Figure 10). The RBCDCs (EGF + Ctx) group exhibited the greatest epidermal thickness (88.40 ± 8.08 μm, SEM), which was significantly higher than that of the Ctrl group (19.91 ± 8.29 μm, SEM) (*** *p* < 0.001) and the corresponding FF (EGF + Ctx) group (### *p* < 0.001). These findings indicate that targeted delivery of EGF via RBC-derived carriers markedly enhanced re-epithelialization compared with administration of the free drug.

In contrast, the FF (EGF + Ctx) group exhibited an epidermal thickness of 16.41 ± 4.29 μm, which did not differ significantly from the Ctrl group (ns). This finding suggests that administration of EGF in its free form did not substantially improve epidermal regeneration under the conditions of the present study. The RBCDCs (Ctx) group demonstrated an intermediate epidermal thickness (46.12 ± 14.65 μm), which was not significantly different from that of the Ctrl group (ns). However, epidermal thickness was significantly greater than in the corresponding FF (Ctx) group (# {*p* < 0.05}), indicating that erythrocyte-mediated delivery of ceftriaxone moderately improved epidermal regeneration compared with the free drug. Similarly, the FF (Ctx) group exhibited the lowest epidermal thickness (7.66 ± 1.33 μm) and showed no statistically significant difference compared with the Ctrl group (ns), suggesting that free ceftriaxone alone had only a limited effect on epidermal regeneration.

Collectively, these results demonstrate that RBCDC-mediated delivery, particularly of the combined EGF and ceftriaxone formulation, substantially promoted re-epithelialization compared with the corresponding free-drug formulations, highlighting the therapeutic advantage of erythrocyte-based targeted drug delivery for promoting wound healing.

### 3.9. Quantitative Assessment of Granulation Tissue Depth

**Figure 11:**
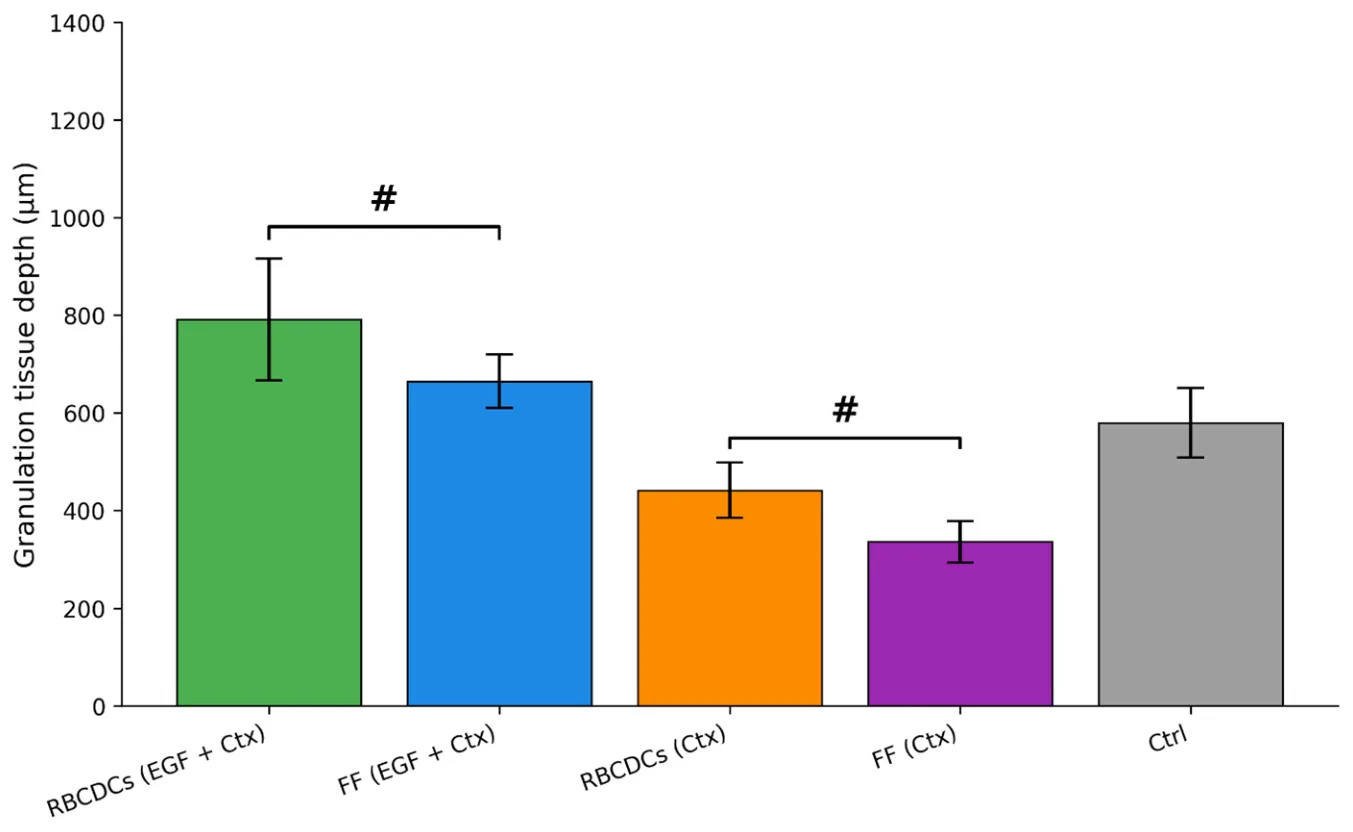
Quantitative assessment of granulation tissue depth on day 9 after wound induction. Granulation tissue depth was measured in wound sections from all experimental groups. Data are presented as the mean ± SEM (*n* = 6 per group). Statistical analysis was performed using one-way ANOVA followed by Dunnett’s post hoc test to compare each experimental group with the control (Ctrl) and Tukey’s multiple comparisons test for pairwise comparisons between experimental groups. # *p* < 0.05 for the indicated pairwise comparisons according to Tukey’s multiple comparisons test.

On day 9 after wound induction, the depth of granulation tissue was quantitatively assessed in all experimental groups (Figure 11).

The greatest granulation tissue depth was observed in the RBCDCs (EGF + Ctx) group (791.2 ± 125.1 μm), followed by the FF (EGF + Ctx) group (664.6 ± 54.4 μm), the Ctrl group (579.7 ± 71.0 μm), the RBCDCs (Ctx) group (441.7 ± 56.9 μm), and the FF (Ctx) group (336.1 ± 42.5 μm) (Figure 11). One-way ANOVA followed by Tukey’s multiple comparisons test demonstrated that the granulation tissue depth was significantly greater in the RBCDCs (EGF + Ctx) group than in the FF (EGF + Ctx) group (*p* = 0.043). Similarly, the RBCDCs (Ctx) group exhibited a significantly greater granulation tissue depth than the FF (Ctx) group (*p* = 0.028). However, Dunnett’s post hoc test revealed no statistically significant differences between any experimental group and the Ctrl group (*p* > 0.05).

These findings indicate that encapsulation of EGF and ceftriaxone, as well as ceftriaxone alone, into RBC-derived carriers enhanced granulation tissue formation compared with administration of the corresponding free drugs, suggesting that RBCDC-mediated delivery promotes tissue regeneration during diabetic wound healing.

### 3.10. Quantitative Assessment of Collagen Deposition

**Figure 12:**
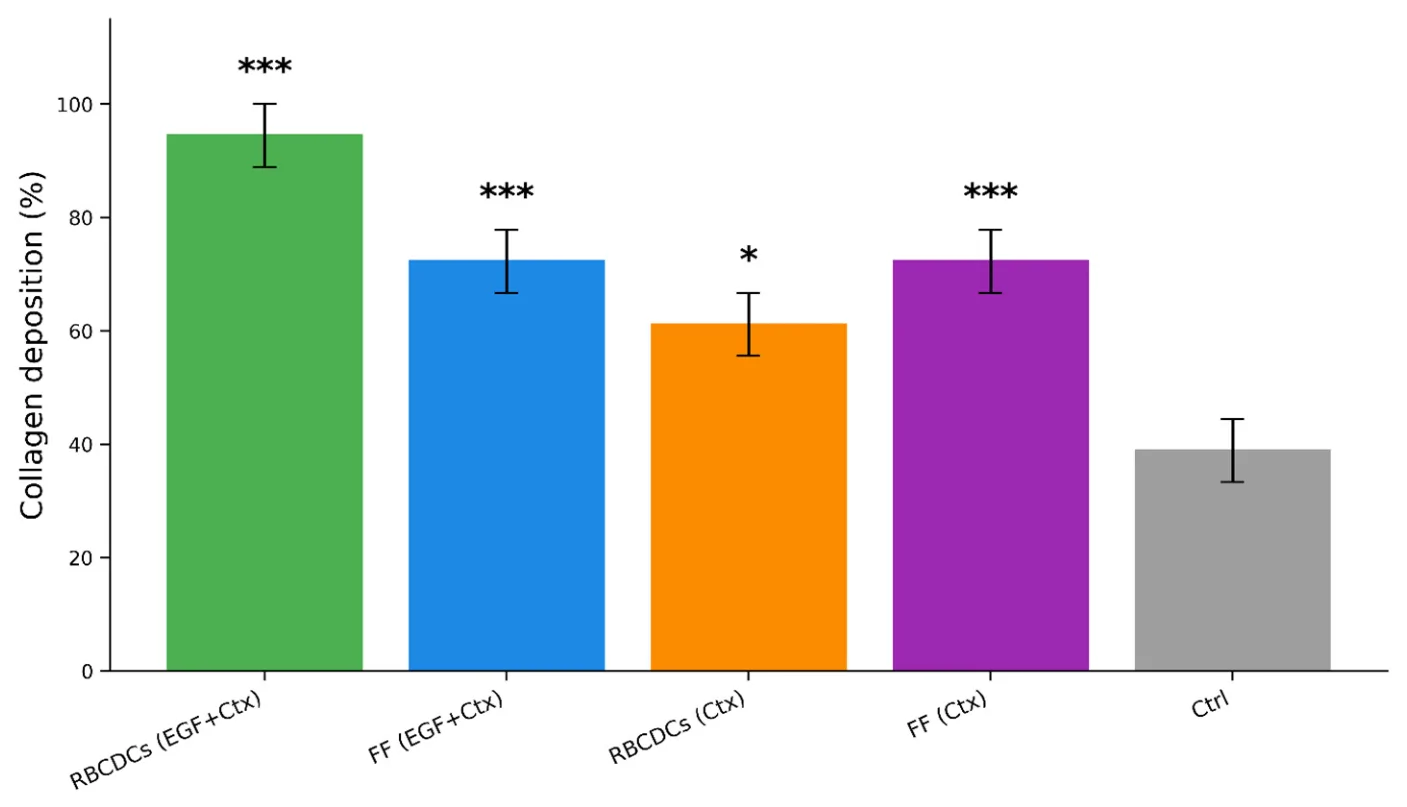
Quantitative assessment of collagen deposition on day 9 after wound induction. Collagen deposition was evaluated using a semi-quantitative histological scoring system (0–3), with the maximum score normalized to 100%. Data are presented as the mean ± SEM (*n* = 6 per group). Statistical analysis was performed using one-way ANOVA followed by Dunnett’s multiple comparisons test to compare each experimental group with the Ctrl group. Compared with the control group, collagen deposition was significantly increased in the FF (Ctx) (*** *p* < 0.001), FF (EGF + Ctx) (*** *p* < 0.001), RBCDCs (Ctx) (* *p* = 0.030), and RBCDCs (EGF + Ctx) (*** *p* < 0.001) groups. Statistical significance compared with the control group is indicated as * *p* < 0.05 and *** *p* < 0.001.

Collagen deposition was quantitatively evaluated on day 9 using a semi-quantitative histological scoring system (0–3), where the maximum score corresponded to 100% collagen deposition (Figure 12).

The RBCDCs (EGF + Ctx) group exhibited the highest collagen deposition score (94.45 ± 5.55%, SEM), followed by the FF (EGF + Ctx) and FF (Ctx) groups (both 72.25 ± 5.55%), the RBCDCs (Ctx) group (61.13 ± 5.57%), and the Ctrl group (38.87 ± 5.57%). Dunnett’s multiple comparisons test revealed that collagen deposition was significantly greater in all treatment groups than in the Ctrl group. Specifically, collagen deposition was significantly increased in the RBCDCs (EGF + Ctx) (*p* < 0.001), FF (EGF + Ctx) (*p* < 0.001), RBCDCs (Ctx) (*p* = 0.030), and FF (Ctx) (*p* < 0.001) groups.

Among all treatment groups, the RBCDCs (EGF + Ctx) formulation achieved the highest level of collagen deposition, indicating enhanced extracellular matrix remodeling and more advanced tissue maturation. These findings suggest that targeted delivery of EGF and ceftriaxone using autologous RBC-derived carriers promotes collagen synthesis and accelerates the remodeling phase of diabetic wound healing.

### 3.11. Quantitative Assessment of Angiogenesis

**Figure 13:**
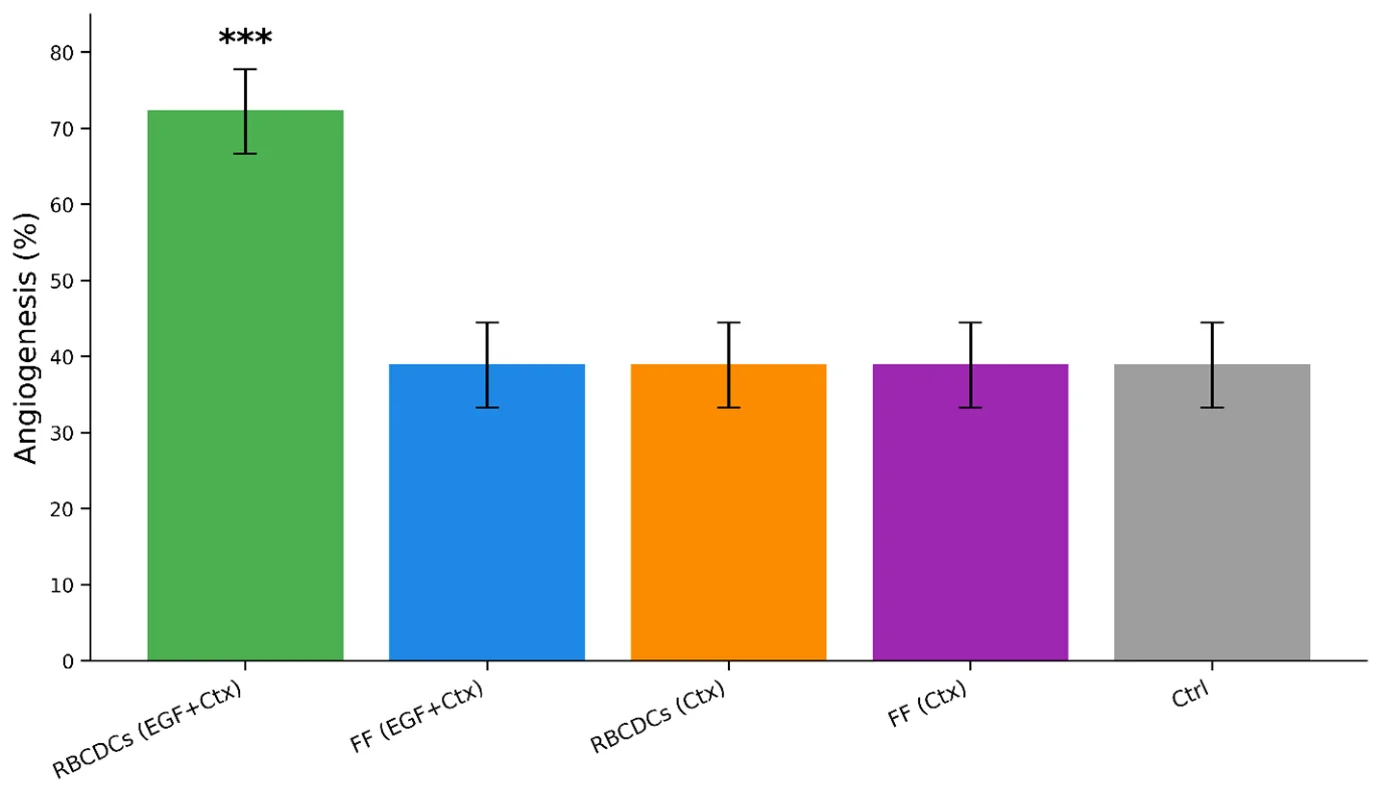
Quantitative assessment of angiogenesis on day 9 after wound induction. Angiogenesis was evaluated using a semi-quantitative histological scoring system (0–3), with the maximum score normalized to 100%. Data are presented as the mean ± SEM (*n* = 6 per group). Statistical analysis was performed using one-way ANOVA followed by Dunnett’s multiple comparisons test to compare each experimental group with the Ctrl group. Only the RBCDCs (EGF + Ctx) group showed a significantly higher angiogenesis score than the Ctrl group (*** *p* < 0.001), whereas no significant differences were observed for the FF (Ctx), FF (EGF + Ctx), or RBCDCs (Ctx) groups. Statistical significance compared with the control group is indicated as *** *p* < 0.001.

Angiogenesis was quantitatively evaluated on day 9 using a semi-quantitative histological scoring system (0–3), with the maximum score normalized to 100% (Figure 13). The RBCDCs (EGF + Ctx) group demonstrated the highest angiogenesis score (72.25 ± 5.55%, SEM), whereas the FF (EGF + Ctx), RBCDCs (Ctx), FF (Ctx), and Ctrl groups all exhibited identical angiogenesis scores of 38.87 ± 5.57%.

Statistical analysis demonstrated that only the RBCDCs (EGF + Ctx) group exhibited a significantly greater level of angiogenesis than the Ctrl group (*p* < 0.001). No statistically significant differences were observed between the FF (EGF + Ctx), RBCDCs (Ctx), or FF (Ctx) groups and the Ctrl group (*p* > 0.05). These findings indicate that targeted delivery of EGF in combination with ceftriaxone using autologous RBC-derived carriers significantly enhanced neovascularization, whereas administration of the corresponding free-drug formulations or RBCDC-mediated delivery of ceftriaxone alone did not produce a comparable pro-angiogenic effect. Enhanced angiogenesis likely contributed to the improved tissue regeneration and accelerated wound healing observed in the RBCDCs (EGF + Ctx) group.

### 3.12. EGF Concentrations in Wound Tissues Following Treatment

**Figure 14:**
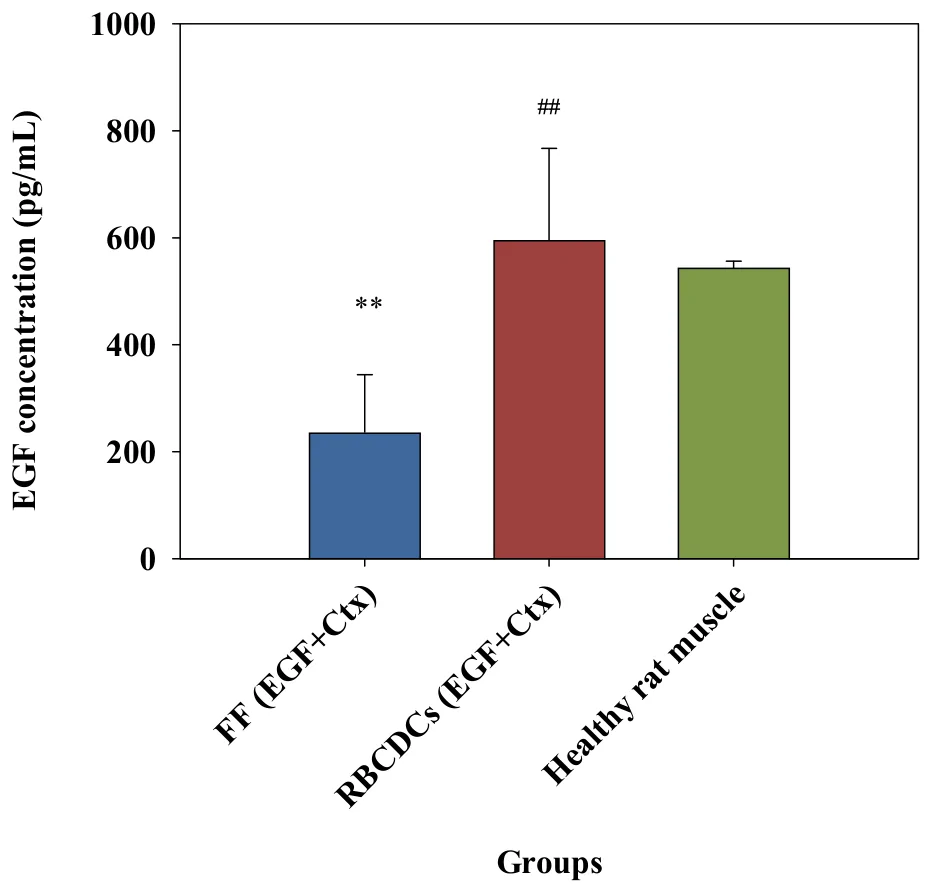
Tissue concentration of EGF on day 9 after treatment in the FF (EGF + Ctx) and RBCDCs (EGF + Ctx) groups, compared to the baseline EGF level in healthy rat muscle. Data are presented as mean ± SEM. Statistical analysis was conducted using One-way ANOVA to assess differences between three independent groups, followed by Dunnett’s post hoc test, where each experimental group was compared to the corresponding healthy rat muscle concentration. A *p*-value of less than 0.05 was considered statistically significant. Statistical significance is indicated as ** *p* < 0.01. Statistical significance between RBCDCs and their corresponding free formulations is indicated as ## *p* < 0.01, for comparisons of RBCDCs (EGF + Ctx) vs. FF (EGF + Ctx).

EGF concentrations were measured in wound tissues from the FF (EGF + Ctx) and RBCDCs (EGF + Ctx) groups and compared with the physiological EGF level in healthy rat muscle. The EGF concentrations in wound tissues on day 9 after treatment are shown in Figure 14.

On day 9 after treatment, the highest tissue EGF concentration was observed in the RBCDCs (EGF + Ctx) group (595.6 ± 78.4 pg/mL), which did not differ significantly from the physiological EGF level in healthy rat muscle (544.0 ± 15.2 pg/mL, *p* = 0.705). In contrast, the FF (EGF + Ctx) group exhibited a significantly lower tissue EGF concentration (236.2 ± 41.6 pg/mL), which was significantly below that of healthy rat muscle (*p* = 0.01). Direct comparison between the two treatment groups demonstrated that tissue EGF concentrations were significantly higher in the RBCDCs (EGF + Ctx) group than in the corresponding FF (EGF + Ctx) group (*p* < 0.01). These findings indicate that erythrocyte-mediated delivery markedly improves the retention and accumulation of EGF within wound tissues compared with administration of the free growth factor.

## 4. Discussion

This study demonstrates that autologous RBC-derived carriers markedly enhance the therapeutic efficacy of EGF and ceftriaxone in diabetic wound healing. Compared with the corresponding free-drug formulations, RBCDC-mediated delivery accelerated wound closure, promoted earlier resolution of inflammation, enhanced re-epithelialization, stimulated collagen deposition and angiogenesis, and maintained tissue EGF concentrations close to physiological levels. Collectively, these findings indicate that RBCDCs provide an effective biomimetic platform for sustained local delivery of growth factors and antibiotics in diabetic wounds.

Recombinant growth factors are recognized as key regulators of tissue repair because they stimulate cell migration, proliferation, differentiation, angiogenesis, and extracellular matrix remodeling. Topical application of CTGF/CCN2 has shown effectiveness in promoting diabetic wound healing in rodent models compared to controls [17]. Nevertheless, their therapeutic application remains limited by rapid proteolytic degradation, short biological half-lives, and poor retention within chronic wound exudates. Consequently, repeated administration or high doses are often required to maintain therapeutic activity, increasing treatment costs while reducing overall efficacy.

Among these growth factors, epidermal growth factor (EGF) has been extensively investigated because of its central role in keratinocyte proliferation, fibroblast activation, collagen synthesis, angiogenesis, and re-epithelialization. As a key regulator of wound repair, EGF stimulates the proliferation, migration, and differentiation of skin cells and has demonstrated considerable therapeutic potential in diabetic wounds [11,55,56,57,58,59,60,61]. Clinical studies have further shown that recombinant human EGF reduces healing time and amputation rates in patients with diabetic foot ulcers. However, the clinical benefits of EGF remain constrained by its limited stability and rapid elimination from the wound environment. To overcome these limitations, numerous delivery systems—including hydrogels, hyaluronic acid conjugates, polymeric scaffolds, liposomes, nanoparticles, and other biomaterials—have been developed to improve EGF stability and prolong local retention. For example, a novel EGF–hyaluronic acid (HA) conjugate protects EGF from enzymatic degradation and enhances tissue penetration, thereby improving its transdermal delivery and therapeutic efficacy [29]. Furthermore, a nanoparticle-based delivery system has been developed to achieve the sustained co-delivery of angiogenic growth factors, including VEGF and bFGF, together with the antimicrobial peptide K4 [37]. This platform provides high encapsulation efficiency and sustained release of the therapeutic agents, thereby promoting angiogenesis while simultaneously exhibiting broad-spectrum antibacterial activity against both Gram-positive and Gram-negative bacteria. Joh et al. reported that the concentration of EGF in human skin and muscle tissues varies depending on the tissue type and the individual’s health status. Enzyme immunoassay revealed that the serum EGF concentrations in healthy males and females were 780 pg/mL and 604 pg/mL, respectively [62]. A study evaluating human skeletal muscle-derived supernatant (not intact tissue) found via ELISA that bFGF was present at 325.6 pg/mL (± 210.2 pg/mL) in muscle extract fluid, though this does not reflect native tissue concentration in situ. Baseline (homeostatic) concentrations of EGF and FGF in uninjured rat skeletal muscle are rarely quantified in the literature, as they are often extremely low or fall below detection limits. While several studies report increased expression or protein levels in response to injury, inflammation, or exogenous growth factor treatment, they do not include ELISA-based quantitative measurements in healthy muscle tissue. Most available data are derived from immunohistochemistry (IHC), mRNA ex-pression analysis, or relative fold-change comparisons following injury or treatment [63,64,65].

Despite these advances, many synthetic delivery systems remain limited by manufacturing complexity, potential immunogenicity, regulatory challenges, and high production costs. In contrast, autologous RBC-derived carriers exploit physiological macrophage-mediated drug delivery. The complementary therapeutic roles of these two agents in this study are as follows: EGF promotes tissue repair and wound regeneration, whereas ceftriaxone provides antibacterial activity, thereby addressing both the regenerative and infectious components of diabetic wound healing. Based on the proposed mechanism of RBCDC-mediated drug delivery, locally administered drug-loaded erythrocytes are expected to undergo gradual phagocytosis by activated macrophages, resulting in sustained intracellular release of encapsulated therapeutics within the inflammatory microenvironment. This biological delivery mechanism prolongs local drug exposure while minimizing systemic distribution and distinguishes RBCDCs from conventional synthetic carriers.

The present findings strongly support this mechanism. Cytological and histological analyses demonstrated that RBCDC-mediated delivery accelerated the transition from the inflammatory to the proliferative phase of wound healing. Earlier resolution of neutrophilic inflammation, increased macrophage and fibroblast activity, and enhanced extracellular matrix deposition indicate a more favorable regenerative microenvironment. These changes were accompanied by faster wound closure and earlier tissue remodeling, suggesting that sustained local release of EGF effectively coordinates multiple stages of the healing process. Histomorphometric analysis further confirmed the regenerative advantages of RBCDC-mediated delivery. The RBCDCs (EGF + Ctx) group exhibited the greatest epidermal thickness, indicating markedly enhanced re-epithelialization. Likewise, collagen deposition was significantly greater than in the control group, demonstrating accelerated extracellular matrix remodeling and wound maturation. Importantly, only the RBCDCs (EGF + Ctx) group exhibited significantly enhanced angiogenesis, indicating that sustained local availability of EGF is required to stimulate effective neovascularization. The absence of a comparable angiogenic response following administration of free EGF further emphasizes the importance of prolonged growth factor retention rather than simply increasing the administered dose. Collectively, these findings demonstrate coordinated activation of multiple regenerative processes rather than improvement of a single histological parameter.

An additional mechanistic finding of the present study was the sustained tissue retention of EGF achieved by RBCDC-mediated delivery. Unlike free EGF, which is rapidly degraded and removed from the wound environment, encapsulated EGF maintained tissue concentrations approaching physiological levels observed in healthy rat muscle. This prolonged local retention provides a plausible explanation for the enhanced re-epithelialization, collagen deposition, angiogenesis, and granulation tissue maturation observed in the RBCDC-treated animals. Sustained local availability of EGF likely promoted continuous activation of fibroblasts, keratinocytes, and endothelial cells throughout the proliferative phase of wound healing.

Compared with previously reported growth factor delivery platforms, RBCDCs offer several unique advantages. Because they are prepared from autologous erythrocytes, they exhibit excellent biocompatibility, complete biodegradability, minimal immunogenicity, and physiological compatibility. Furthermore, the present study demonstrates that erythrocyte-derived carriers can simultaneously deliver both a regenerative growth factor and an antimicrobial agent while improving multiple aspects of diabetic wound healing. This dual-delivery strategy may be particularly advantageous for diabetic foot ulcers, where impaired tissue regeneration frequently coexists with bacterial infection.

From a translational perspective, RBCDC-mediated delivery may offer several clinical advantages for diabetic wound management. Sustained local delivery of EGF together with ceftriaxone has the potential to reduce dosing frequency, maintain therapeutic drug concentrations within infected wounds, improve healing outcomes, and decrease systemic antibiotic exposure. Although further studies are required to evaluate long-term safety, pharmacokinetics, and clinical efficacy, these findings support continued development of RBCDC-based therapeutics for chronic diabetic wounds.

This study has several limitations. First, experiments were performed in a rat model, and translation of these findings to human diabetic wounds requires further investigation. Second, only a single combination of growth factor and antibiotic was evaluated. Third, long-term functional outcomes, scar quality, and biomechanical properties of the regenerated tissue were not assessed. Finally, the molecular mechanisms governing macrophage-mediated intracellular release of encapsulated EGF require further investigation.

In conclusion, encapsulation of EGF and ceftriaxone within autologous RBC-derived carriers significantly enhanced diabetic wound healing by accelerating resolution of inflammation, promoting re-epithelialization, stimulating angiogenesis and collagen deposition, and maintaining sustained local EGF concentrations. These findings identify RBCDCs as a promising biomimetic delivery platform for targeted delivery of regenerative therapeutics and support their further development for the treatment of chronic diabetic wounds. Despite such advances, the complex pathophysiology of wound healing in diabetic patients and the limited clinical use of topical immunomodulatory therapies highlight the need for further research. A deeper understanding of the local anti-inflammatory mechanisms of growth factors is essential to improve diabetic wound healing. A further limitation of the present study is the absence of unloaded RBCDCs, free EGF alone, and EGF-loaded RBCDCs without ceftriaxone. Therefore, the independent contributions of the erythrocyte-derived carrier, EGF, and ceftriaxone cannot be fully distinguished. Accordingly, the therapeutic benefit observed in the RBCDCs (EGF + Ctx) group should be interpreted as the effect of the combined treatment regimen rather than as evidence of an isolated effect of erythrocyte-mediated EGF delivery.

## 5. Conclusions

Autologous red blood cell-derived carriers (RBCDCs) loaded with epidermal growth factor (EGF) and ceftriaxone significantly enhanced diabetic wound healing by providing sustained, targeted local delivery of regenerative and antimicrobial agents. Compared with the corresponding free-drug formulations, RBCDC-mediated delivery accelerated the resolution of inflammation, promoted earlier granulation tissue formation, enhanced re-epithelialization, stimulated collagen deposition and angiogenesis, and maintained tissue EGF concentrations near physiological levels. Cytological, histological, histomorphometric, and ultrastructural analyses consistently demonstrated earlier fibroblast activation, accelerated extracellular matrix remodeling, and more advanced wound maturation following RBCDC treatment.

Among all treatment regimens, the RBCDCs (EGF + Ctx) group showed the most pronounced regenerative response, including faster wound closure, earlier transition from the inflammatory to the proliferative phase of healing, and more advanced tissue regeneration compared with the corresponding free-drug formulations. However, because unloaded RBCDCs and EGF-only control groups were not included in the experimental design, the independent contributions of EGF and the erythrocyte-derived carrier cannot be fully distinguished. Therefore, the observed therapeutic benefit should be interpreted as the effect of the combined RBCDC-based treatment regimen rather than as evidence of an isolated effect of erythrocyte-mediated EGF delivery.

Overall, the present findings support autologous RBCDCs as a promising biocompatible and biomimetic platform for the local administration of regenerative and antimicrobial agents. The improved wound-healing outcomes observed with the RBCDC-based treatment regimens warrant further investigation of this approach in diabetic wound therapy. However, dedicated studies of local pharmacokinetics, systemic drug exposure, carrier stability, and long-term safety are required before conclusions regarding sustained delivery or reduced systemic exposure can be established.

## Figures and Tables

**Table 1:** Dynamics of Wound Healing expressed as a percentage (%) of initial wound size (mean ± SEM, *n* = 6 per group).

	RBCDCs (EGF + Ctx)	FF (EGF + Ctx)	RBCDCs (Ctx)	FF (Ctx)	Ctrl
0 day	0.0 ± 0.0%	0.0 ± 0.0%	0.0 ± 0.0%	0.0 ± 0.0%	0.0 ± 0.0%
3 day	17.7 ± 1.2%	18.7 ± 0.9%	20.3 ± 1.2%	17.0 ± 3.2%	19.3 ± 2.0%
5 day	43.3 ± 3.5%	42.0 ± 3.0%	39.7 ± 1.5%	32.0 ± 3.2%	33.3 ± 1.8%
7 day	100.0 ± 0.0%	58.0 ± 1.5%	55.0 ± 1.2%	48.7 ± 2.3%	56.7 ± 2.0%
9 day	100.0 ± 0.0%	78.3 ± 3.8%	72.3 ± 1.5%	75.0 ± 3.1%	78.7 ± 3.8%

## Data Availability

The original contributions presented in this study are included in the article. Further inquiries can be directed to the corresponding authors.

## Abbreviations

The following abbreviations are used in this manuscript:

RBCDCs	Red blood cell-derived carriers
DFUs	Diabetic foot ulcers
TNF-α	Tumor necrosis factor α
IL-6	Interleukin-6
IL-1β	Interleukin-1β
IFN-γ	Interferon-gamma
IL-10	Interleukin-10
EGF	Epidermal growth factor
PDGF	Platelet-derived growth factor
TGF-β1	Transforming growth factor-beta1
VEGF	Vascular endothelial growth factor
FGF	Fibroblast growth factor
FF (EGF + Ctx)	free form of EGF and Ceftriaxone
RBCDCs (EGF + Ctx)	RBCDCs loaded with EGF and Ceftriaxone
FF(Ctx)	free form of Ceftriaxone
RBCDCs (Ctx)	RBCDCs loaded with Ceftriaxone
RBCs	Red blood cells
SEM	Scanning electron microscopy
TEM	Transmission electron microscopy
LM	Light microscopy
Ctx	Ceftriaxone
Ctrl	Control group
SD	Standard deviation
STZ	Streptozotocin
DMP	DERMO PUNCH skin biopsy system
IACUC	Institutional Animal Care and Use Committee
HPLC	High-performance liquid chromatography
mean ± SD	Mean ± standard deviation
ANOVA	Analysis of variance
IACUC	Institutional Animal Care and Use Committee
mean ± SD	Mean ± standard deviation
HPLC	High-performance liquid chromatography
